# Deciphering unusually large modulations in two related organic hy­droxy channel structures

**DOI:** 10.1107/S2052520626004932

**Published:** 2026-06-10

**Authors:** Arie van der Lee, Ioan Stroia, Li-Bo Huang, Erol Licsandru, Istvan Kocsis, Mihail Deleanu, Christian Hübschle, Mihail Barboiu, Olivier Pérez

**Affiliations:** a Institut Européen des Membranes Université de Montpellier Montpellier France; b Bruker AXS,Östliche Rheinbrückenstr. 49, 76187Karlruhe, Germany; c CRISMAT, CNRS Normandie University ENSICAEN UNICAEN Caen France; Institute of Physics, CAS, Czechia

**Keywords:** modulated structure, organic structure, Lissajous representation, modulation strength

## Abstract

The incommensurately modulated structures of two related organic hy­droxy-channel compounds [(*R*)-1-(1,3-dihydroxypropan-2-yl)-3-(octan-2-yl)urea, **1**; 1-(1,3-dihydroxypropan-2-yl)-3-octylurea, **2**] have been determined and compared to the structure of a non-modulated reference compound [(*R*)-bis-3-(octan-2-yl)urea, **3**] lacking hy­droxy-channel forming groups. The origin of the modulation is explored through the competition between strong orthogonal urea–urea and hy­droxy–hy­droxy interactions, though this competition may not be a prerequisite for modulation. Notably, the modulation in these two compounds is unusually pronounced, as shown by the first-order satellite reflections exhibiting intensities comparable to those of the main reflections. This observation is contextualized by comparing the relative intensity differences between main and satellite reflections in a benchmark set of 117 modulated structures. Additionally, an alternative graphical method is introduced to visualize structural modulation within the basic unit cell. This method combines a conventional ball-and-stick representation of the average structure with Lissajous curves that map the spatial modulation onto the unit cell. The curves are color-coded according to the modulation *t*-parameter, enabling immediate visualization of in-phase and out-of-phase individual modulation displacements.

## Abstract 2

Two related organic hy­droxy-channel compounds are shown to have unusually large structural modulations.

## 1. Introduction

Incommensurately modulated structures are often thought to be found only in inorganic structures with some notable exceptions in organic phases such as thio­urea (Yamamoto, 1980 ▸; Zuñiga *et al.*, 1989 ▸), bi­phenyl, 2-phenyl­benzimidazole (Cailleau, Moussa & Mons, 1979 ▸; Baudour & Sanquer, 1983 ▸; Cailleau, Baudour & Zeyen, 1979 ▸) and host–guest urea inclusion compounds (Forst *et al.*, 1987 ▸; Van Smaalen & Harris, 1997 ▸; Mariette *et al.*, 2022 ▸). Schoenleber wrote in his review on organic molecular compounds with modulated crystal structures that this view might be biased (Schoenleber, 2011 ▸), but did not take completely away the illusion that incommensurateness in organic compounds is more rare than in inorganic compounds (Bakus *et al.*, 2013 ▸). A recent survey on modulated structures published in IUCr journals showed that only 13 from the 102 structures could be classified as organic (Gaydamaka & Rashchenko, 2024 ▸). From the numbers of crystal structures in the main organic [Cambridge Structural Database (CSD): 1300k; November 2025] and inorganic databases (Inorganic Crystal Structure Database, 290k; November 2025) one may conclude that more organic crystals have been determined than inorganic ones, which would make the relative propensity of incommensurateness in inorganic structures still more remarkable. One reason for the high propensity of inorganic structures for incommensurateness could be that the presence of satellite reflections in their diffraction pattern is more easily detected than those in the patterns of organic structures, because of the stronger scattering power of the elements usually present in inorganic structures. The intensities of satellite reflections are usually considered to be weaker than those of the main reflections corresponding to the average structure. Another reason may be that diffraction platforms work more in semi-automated high-throughput mode for organic and organometallic compounds than for inorganic compounds. At the same time, expertise in aperiodic crystallography remains confined to a relatively small community. As a result, diffraction signatures of incommensurate modulation may not always be recognized during routine screening and structure-solution workflows. The identification of such features often requires careful inspection of reciprocal-space sections and familiarity with the characteristic patterns of aperiodicity. Without such examination, automatic procedures may fail to correctly index a diffraction pattern with satellite reflections at irrational positions. Consequently, the crystal may be discarded for further analysis. The present examples illustrate how the attentive examination of reciprocal-space data can reveal modulation features that might otherwise remain unnoticed in routine workflows. Without careful examination of the reciprocal lattice, automatic indexing may indeed fail to distinguish between a single crystal displaying satellite reflections at irrational positions and a multigrain sample. Consequently, many modulated organic structures could remain unrecognized—or be discarded altogether—unless examined closely for satellites. Yet, modern commercial software does, however, give most of the tools needed to recognize and process diffraction data with incommensurate features.

**Figure 1:**
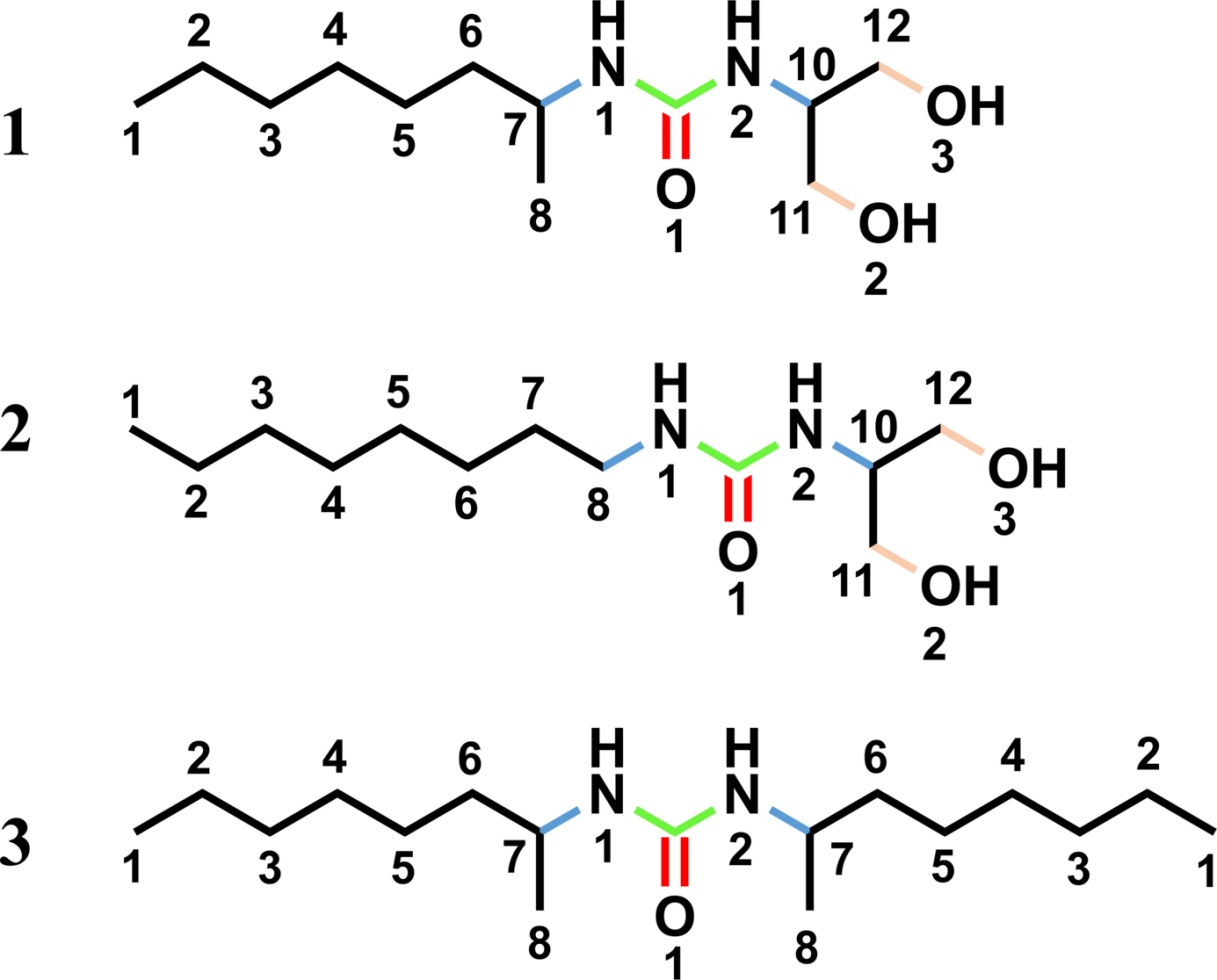
Schemes of **1**, **2** and **3** with atom labeling and color codes for intramolecular bonds to be used in Fig. 10 ▸ and Fig. 14 ▸.

We present in this study two new related incommensurately modulated organic structures (**1** and **2**, see schemes in Fig. 1 ▸) of which **2** was published before by some of the authors of this paper as a triclinic *Z*′ = 9 structure (labeled H4) (Huang *et al.*, 2021 ▸). It was already recognized in the ESI of that paper that the structure might be (in)commensurately modulated, but that there had not been success in treating it as such. The suspicion that the structure was probably incommensurate resulted from the knowledge gathered by the structure solution of **1** in November 2017. The diffraction data of **1** were presented by one of the authors at a workshop of the French network for professional crystallographers (ЯÉCIPROCS) in Lille, France, during a session entitled ‘Structures resisting to everything’. One of the co-authors in this event accepted the challenge and determined and approximately refined the principal features of the incommensurate structure of **1** during the weeks after the workshop, but the structure was never published. We decided to pick up the determination of the incommensurate structure of **2** earlier this year and succeeded in solving and refining it. We observed that the difficulties related to the correct interpretation of the diffraction patterns are, at least in these cases, not related to the weakness of the satellite reflections, but rather to the strength of them, especially for **2**. The first and second-order satellite reflections of latter phase have approximately the same mean intensity as the main reflections, making it very hard to correctly identify the unit cell of the average structure (*i.e.* the basic unit cell) and to do the indexing of the satellite reflections correctly. The relative important strength of the satellite reflections means that the structural modulation is particularly strong in both phases.

The structure solution and refinement of **1** and **2** bear some resemblance with that of quininium (*R*)-mandelate (Schönleber & Chapuis, 2004 ▸), a structure with can be described as a structure with an unusual large value of *Z*′, 15 or 18, depending on the commensurate approximation of the supercell. As for the structure of quininium (*R*)-mandelate, the average structures of **1** and **2** could not be solved from the subset of main reflections only. The reason for the modulation in **1** and **2** is similar to that in quininium (*R*)-mandelate, i.e a competition between intermolecular hydrogen bonds and intramolecular forces that are responsible for the molecular conformation. We also report the structure of a related compound **3** (Fig. 1 ▸) which was accidently crystallized when trying to synthesize **1**.

Both self-assembled compounds **1** and **2** are hy­droxy-channel structures based on octyl-ureido-polyols, which may – when embedded in liposome membranes – transport water with transport rates slightly below rates observed in natural aqua­porins (Agre, 2004 ▸) while at the same time presenting almost total ion rejection. This makes them useful as artificial water channels for the fabrication of membranes for seawater desalination. The single-channel permeability of the channels formed by **2** was the highest (2.33 × 10^8^ water / channel / second) of the six reported structures bearing different hy­droxy­lic interacting heads. The hy­droxy channels inside the crystalline structures as determined by the X-ray diffraction study preclude, however, any water transport, since they are completely dense without any pores or pockets to accommodate water molecules. The observed high transport rates could only be explained by a re-arrangement of the crystal structure into a spongelike mesostructure or a cylindrical pore structure as observed by molecular dynamics simulations, depending on the concentration of the hy­droxy channels within the membrane (Huang *et al.*, 2021 ▸).

Although improved knowledge about the real crystalline structure of hy­droxy channel compounds does not lead directly to better insights in the transport phenomena of the channels inside the lipid bilayers, it does give a hint about the flexibility of the molecular confirmations in relation to intermolecular hydrogen-bond interactions which do play a role in the more dynamic membrane environment.

## 2. Synthesis

All three compounds have been synthesized following the following general procedure: the amino propane­diol or octan-2-yl amine (10 mmol) was mixed with the corresponding equimolar amount of octyl iso­cyanate, with sonication. The mixture was dissolved in tetra­hydro­furan (thf; 10 ml), ethyl acetate (5 ml) and aceto­nitrile (5 ml). The reaction system was heated to 80 °C for 2 h. The resulting solution was dried with conventional rotary evaporation under vacuum to obtain white powder. The products were washed with methanol and hexane, then were separated by rotary centrifuge. The residual solvent was removed by vacuum desiccator. Compound **3** was obtained in an initial attempt to obtain compound **1**. A second attempt with apparently slightly different starting conditions (temperature, pH, …) resulted in the target compound **1**. No attempt was made to understand the different outcome of the two syntheses.

(*R*)-1-(1,3-Di­hydroxy­propan-2-yl)-3-(octan-2-yl)urea, **1**: ^1^H NMR (400 MHz, DMSO-*d*_6_): δ 0.85 (*t*, *J* = 6.4 Hz, 3H, CH_3_^7^), 0.96 (*d*, *J* = 6.6 Hz, 3H, CH_3_^8^), 1.23–1.30 (*m*, 10 H, CH_2_^6^CH_2_^5^CH_2_^4^CH_2_^3^CH_2_^2^), 3.27–3.36 (*m*, 2H, H^B^ CH_2_^13^), 3.37–3.44 (*m*, 2H, H^A^ CH_2_^13^), 3.45–3.60 (*m*, 2H, CH^1^, CH^12^), 4.63 (*t*, *J* = 5.2 Hz, 2H, OH^14^), 5.56 (*d*, *J* = 7.7 Hz, 1H, NH^11^), 5.84 (*d*, *J* = 8.1 Hz, 1H, NH^9^) p.p.m.; ^13^C NMR (100 MHz, DMSO-*d*_6_): δ 13.91 (CH_3_^7^), 21.44 (CH_3_^8^), 22.02 (CH_2_^6^), 25.44 (CH_2_^3^), 28.68 (CH_2_^4^), 31.26 (CH_2_^5^), 36.90 (CH_2_^2^), 44.58 (CH^1^), 52.62 (CH^12^), 60.53 (CH_2_^13^ C^B^), 60.55 (CH_2_^13^ C^A^), 157.44 (CO^10^); MS: m/z (%): 247.6 (100) [MD-I-3-P + H], 269.7 [MD-I-3-P + Na], 516.2 [2*MD-I-3-P + Na].

1-(1,3-Di­hydroxy­propan-2-yl)-3-octylurea, **2**: ^1^H NMR (DMSO-*d*_6_, 400 MHz): δ (p.p.m.) = 0.86 (*t*, *J* = 6 Hz, 3H, CH_3_CH_2_), 1.21–1.35 [*m*, 12H, CH_3_(CH_2_)_6_CH_2_], 2.94 (*q*, *J* = 6.7 Hz, 2H, CH_2_CH_2_NH), 3.38–3.43 [*m*, 2H, CH(CH_2_OH)_2_], 3.45–3.53 (*m*, 1H, CH_2_CHNH), 4.62 [*t*, *J* = 5.3 Hz, 2H, CH(CH_2_OH)_2_], 5.62 (*d*, *J* = 7.8 Hz, 1H, CH_2_NH), 5.98 (*t*, *J* = 5.6 Hz, 1H, CHNH); ^13^C NMR (DMSO-*d*_6_, 100 MHz): δ (p.p.m.) = 14.44 (CH_3_), 22.57, 26.88, 29.19, 29.24, 30.46, 31.72 (6 × CH_2_), 39.60 (CH_2_NH), 53.09 (CHNH), 60.90 (CH_2_OH), 159.45 (CO); MS Calc. = 246.4, ESI-MS found [M+H^+^]^+^ = 247.4.

(*R*)-Bis-3-(octan-2-yl)urea, **3**: ^1^H NMR (400 MHz, DMSO-*d*_6_): δ 0.86 (*t*, *J* = 6.4 Hz, 3H, CH_3_^7^), 0.96 (*d*, *J* = 6.6 Hz, 3H, CH_3_^8^), 1.23–1.30 (*m*, 10H, CH_2_^6^CH_2_^5^CH_2_^4^CH_2_^3^CH_2_^2^), 5.56 (*d*, *J* = 7.7 Hz, 1H, NH^11^), 5.84 (*d*, *J* = 8.1 Hz, 1H, NH^9^) p.p.m.; ^13^C NMR (100 MHz, DMSO-*d*_6_): δ 13.91 (CH_3_^7^), 21.44 (CH_3_^8^), 22.02 (CH_2_^6^), 25.44 (CH_2_^3^), 28.68 (CH_2_^4^), 31.26 (CH_2_^5^), 36.90 (CH_2_^2^), 44.58 (CH^1^), 157.44 (CO^10^).

## 3. Diffraction experiments and interpretation of the reciprocal lattices

Crystals of **1** and **3**, obtained by slow evaporation from methano­lic solutions, were selected and mounted on a Gemini single-crystal diffractometer equipped with a Sapphire-3 detector. The *CrysAlisPro* program (Rigaku Oxford Diffraction, 2012 ▸) was used to collect data, for frame integration using default parameters, for the empirical absorption correction using spherical harmonics employing symmetry-equivalent and redundant data, and for the correction for Lorentz and polarization effects. ω rotational scans (counting time 57.5 s per frame for **1** and 42.5 s for **3**) were employed for the acquisition of the intensity data. The data collection temperature was 175 K.

Crystals of **2** were tested and measured at the XRD1 beamline of the Elettra synchrotron (Basovizza, Italy) at a wavelength of 0.6999 Å with a Huber four-circles κ-goniometer and a Pilatus-2M (Dectris) detector at 100 K (Lausi *et al.*, 2015 ▸). The data were collected at 100 K in 360 ϕ-scan frames with an exposure time of 1 second for each frame. The data were integrated using the *XDS* software (Kabsch, 2010*b* ▸; Kabsch, 2010*a* ▸) for the supercell approximation and with *CrysAlisPro* (Rigaku Oxford Diffraction, 2012 ▸) for the incommensurate model.

All structural refinements were carried out with *JANA2020* (Petříček *et al.*, 2023 ▸). Table 1 ▸ compiles the crystal data, data collection and refinement parameters for the three structures.

### 3.1. Interpretation of the reciprocal lattice of **1**

Indexing proved to be challenging with less than 70% of the integrated spots assigned to lattices with varying cell parameters in a monoclinic setting with only the *b*-axis constant at about 4.63 Å, notably the following two cells: *a* = 34.9190 Å, *b* = 4.6262 Å, *c* = 35.7164 Å, α = 90.0240°, β = 98.6917°, γ = 89.9655°, *V* = 5703 Å^3^ or *a* = 103.5825 Å, *b* = 4.6268 Å, *c* = 53.6123 Å, α = 90.0558°, β = 91.4504°, γ = 89.9694°, *V* = 25685 Å^3^. It could be anticipated that the cell axis of 4.63 Å corresponds to the intermolecular repeat distance of aligned urea motifs (Nguyen *et al.*, 2001 ▸), but the *a* and *c* parameters of these automatically determined unit cells are unusually large and hence the volumes, suspecting a twinned structure or a commensurately or incommensurately modulated structure. It was furthermore noticed that a substantial number of reciprocal lattice nodes is systematically absent.

Based on the invariance of *b* and the apparent monoclinic symmetry, reconstructions of the (*h*0*l*)*, (*h*1*l*)* and (*h*2*l*)* diffraction planes, perpendicular to the *b* axis, were performed.

A detailed examination of these diffraction patterns reveals an unusual distribution of reflections: broad ribbons of diffraction spots alternate with dark, reflection-free regions. This pattern is strongly reminiscent of intensity distributions typically observed in modulated phases.

Within each reflection ribbons attempts were made to identify periodicities that could allow the diffraction peaks to be assigned in terms of main reflections—corresponding to the average structure which is equal to the zero-order terms of the harmonic expansions of the structural coordinates (*x*_0_,*y*_0_,*z*_0_)—and satellite reflections, which characterize the modulation as a periodic perturbation of this average structure. The main difficulty in identifying the various periodicities lies in the absence of a clear intensity hierarchy among the reflections. The modulation appears to be very strong, making it impossible to distinguish the main and satellite reflections based solely on their intensities. As a result, the solution relies entirely on a geometrical analysis of the diffraction pattern. After numerous trials, we identified a basic unit cell (Table 1 ▸) indexing only 23% of the reflections. The ratio between the volumes of the two before-mentioned supercells and this basic cell is very close to 4 and 18, respectively, implying a direct transformation relation between them.

By introducing a modulation vector **q** = 0.226a*+ 0.508c*, the indexing rate increased to over 80%. The remaining unindexed reflections are either randomly distributed or located on the tails of indexed peaks, suggesting that they correspond to peak broadening rather than distinct reflections. Satellite reflections are observed up to third order.

**Figure 2:**
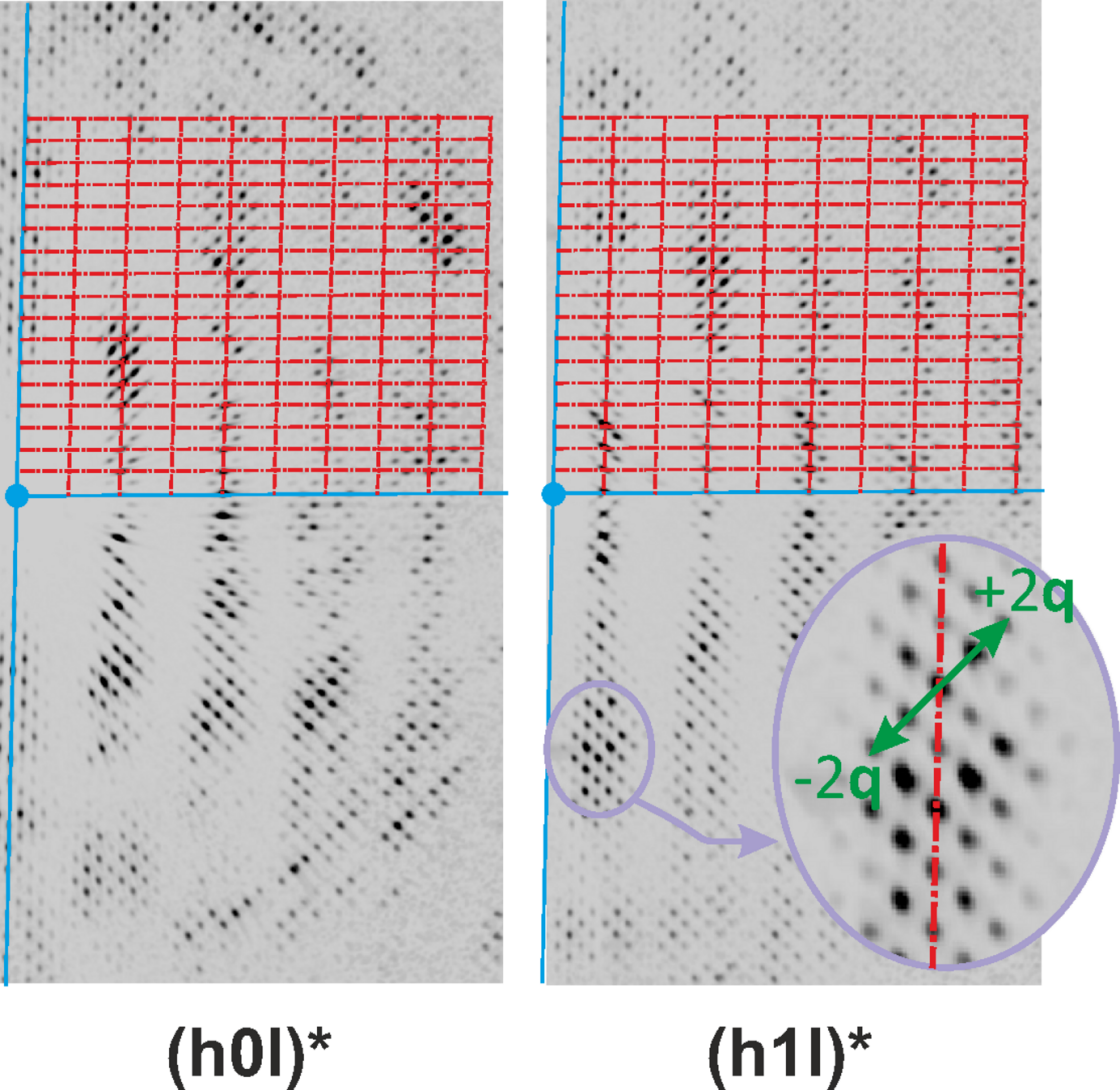
Reconstructed (*h*0*l*)* and (*h*1*l*)* diffraction planes of **1**. The filled blue circle shows the origin of the pattern. The horizontal and almost vertical blue lines are corresponding to the a* and c* direction, respectively. The red dot-dashed lines are drawing the lattice defined by the basic unit cell. The purple-circled magnification highlights the modulation vector.

A comparison of the (*h*0*l*)* and (*h*1*l*)* planes reveals a *C*-centered lattice (Fig. 2 ▸). Data integration was carried out to obtain 31811 reflections, 18579 were independent and 10348 were measured with *I* > 3σ(*I*) yielding *R*_int_ = 3.5%.

The structure could then be solved directly in superspace (Palatinus, 2004 ▸) using *Superflip* (Palatinus & Chapuis, 2007 ▸) with superspace groups *C*2(σ_1_0σ_2_)0 or *C*2/*m*(σ_1_0σ_2_)00. A first structural model was obtained in the non-centrosymmetric superspace group with 12 C, 3 O and 2 N atoms. The positions of 26 H atoms were determined based on geometrical considerations; positional constraints were imposed so that the hydrogen atoms move in accordance with the atomic displacements of the bonded carbon, oxygen, or nitro­gen atoms. Harmonic modulation waves were introduced up to the third order (consistent with the experimental observation of satellite reflections up to the third order) to model the atomic displacement of C, O and N atoms; a harmonic wave was also defined to describe their anisotropic atomic displacement parameters (ADPs).

### 3.2. Interpretation of the reciprocal lattice of **2**

The structure of **2** has already been published as a *Z*′ = 9 structure before (Huang *et al.*, 2021 ▸) and shortly described in supporting information. Here we give additional details about the reciprocal space interpretation, structure solution and refinement as a modulated structure. Consistent cell parameters corresponding to the *Z*′ = 9 structure were obtained for different crystals.

**Figure 3:**
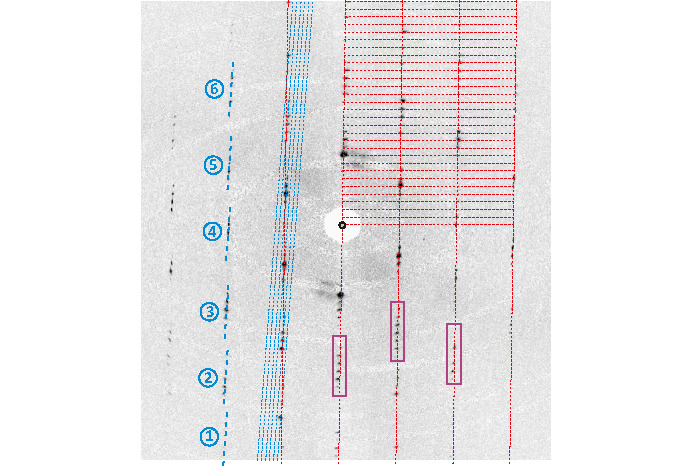
Reconstructed (*hk*0)* plane calculated in the original cell (**a**_s_, **b**_s_, **c**_s_) for **2**. In the right part, (**a**_s_, **b**_s_, **c**_s_) is drawn. Significant deviation of the reflections from the vertical red dashed lines are observed (see purple rectangles). Left part: identification of six segments of reflections. The blue dashed lines highlight the significant deviation in between the segments direction and the red dashed line corresponding to the **b**_s_ stacking direction.

For the re-investigation using the superspace formalism, the diffraction frames were imported into the *CrysAlisPro* package and a closer inspection of the diffraction pattern revealed imperfections in the initial model. The reconstructed (*hk*0)* plane (Fig. 3 ▸) highlights inconsistencies: although the lattice based on the given unit cell is generally followed, certain reflections are misaligned, and large areas devoid of reflections are evident. In addition, segments of reflections appear aligned along the b* direction. These observations suggest that the original cell (***a***_s_, ***b***_s_, ***c***_s_) serves only as an approximate description of the true structure.

**Figure 4:**
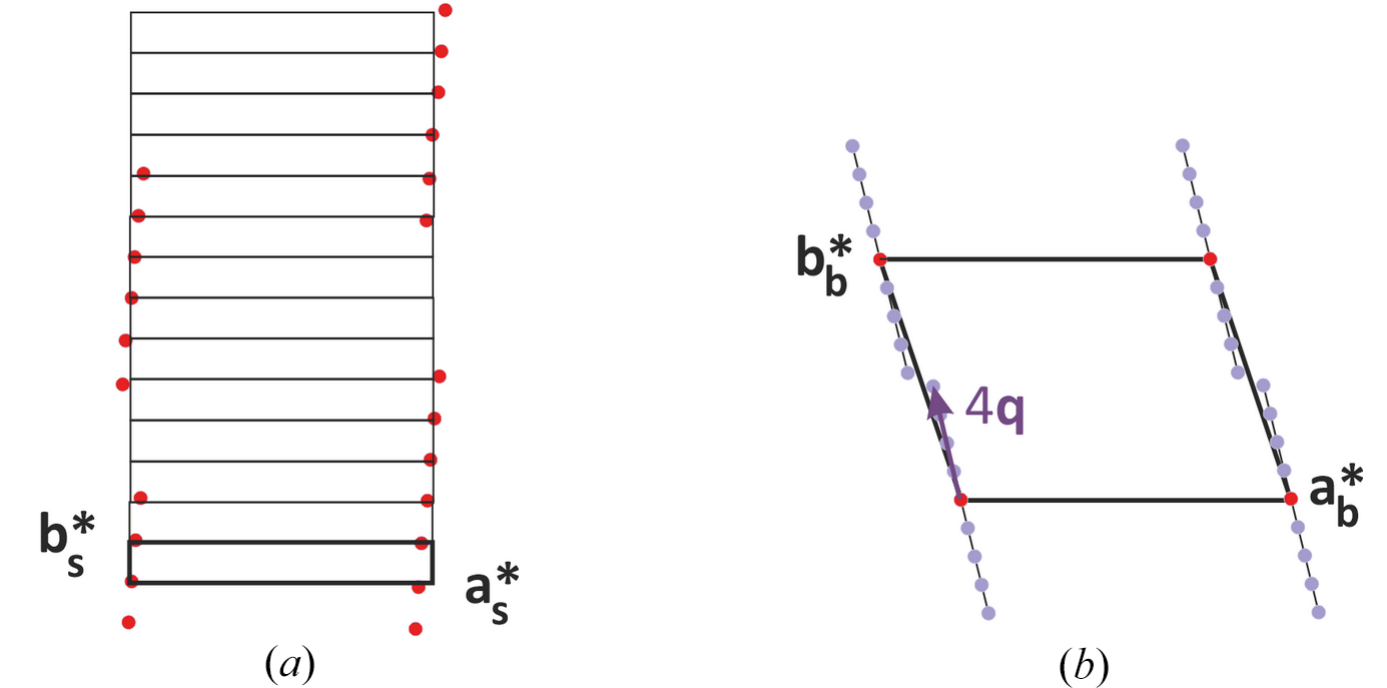
Scaled diffraction diagram of the C_1__2_H_2__6_N_2_O_3_ compound and its interpretation using the unit cells (*a*) (**a**_s_*, **b**_s_*, **c**_s_*) and (*b*) (**a**_b_*, **b**_b_*, **c**_b_*). In (*a*) the reflections are not all accurately accounted for by the unit cell. In contrast, in (*b*), the satellite reflections are properly indexed using the modulation vector **q** = 0.008a* + 0.117b* + 0.579c*. Due to the significant 0.579**c**_b_* component of the modulation vector, these reflections lie outside the plane of the sketch. For clarity, they have been projected onto the (*h*_b_*k*_b_0)* plane.

Based on the midpoints of these reflection segments, a basic unit cell (***a***_b_, ***b***_b_, ***c***_b_) was defined (Table 1 ▸). The volume of this cell is nine time smaller of that of the aforementioned supercell, explaining the description of the original structure as a *Z*′ = 9 structure. This new cell corresponds to a refined lattice, where its nodes pass through the midpoints of the aforementioned segments. Unindexed reflections in the original model can now be interpreted as satellite reflections. Fig. 4 ▸ gives a schematic view of the diffraction patterns with respect to the reciprocal supercell approximation and the reciprocal basic unit cell.

Introducing the modulation wavevector **q** = 0.008**a**_b_* + 0.117**b**_b_* + 0.579**c**_b_*, all reflections—main and satellites—could be successfully indexed. Satellite reflections were observed up to the fourth order. A major challenge in identifying the correct cell stemmed from the comparable intensities of main and satellite reflections. It should be noted that only the high resolution of our dataset collected at Elettra allowed us to identify the weak component according to a* of the modulation vector.

The relatively large atomic displacement parameters (ADPs) refined in both the original and new unit cells, coupled with the irrational components of the modulation vector **q**, support the conclusion that the published structure is an approximation of a more accurate incommensurately modulated model.

The first step in the data processing procedure consisted in the accurate determination of the basic reciprocal lattice and modulation vector parameters. Using the complete set of diffraction images, the positions of all observed reflections were extracted with the *CrysAlisPro* software. To ensure the reliability of the indexing procedure, the dataset was processed independently in parallel using both *CrysAlisPro* and *JANA2020*.

In both programs, the reciprocal basis vectors **a**_b_*, **b**_b_*, **c**_b_*, and the modulation vector ***q*** were determined manually. This procedure proved to be particularly demanding and time-consuming, but was unavoidable in the present case because the comparable intensities of main and satellite reflections prevented an automatic prioritization of reflection classes during indexing. Consequently, careful manual selection and verification of reflection positions were required in order to define a consistent reciprocal grid.

Once a reliable set of reflections had been established, both the unit-cell parameters and the components of the modulation vector were refined against the selected reflections. The results obtained from the two independent software packages were fully consistent, providing confidence in the robustness of the indexing solution. The refined unit cell and modulation vector were subsequently used as fixed input parameters for the data reduction.

The integration step itself presented additional difficulties. The unusually high intensity of the satellite reflections, combined with the very small component of the modulation vector along **a**_b_*, made the refinement of cell and modulation parameters during integration unstable. In particular, unconstrained refinement systematically led to incorrect indexing and inconsistent reflection statistics. To overcome this problem, both the unit-cell parameters and the components of the modulation vector were fixed to the values obtained during the preliminary refinement stage. In addition, the components of ***q*** were kept fixed throughout the integration procedure due to the near-zero value of the **a**_b_* component. This precaution proved essential for achieving a stable and physically meaningful data reduction.

Ultimately, a dataset of 10 854 independent reflections with *I* > 3σ(*I*) was obtained, yielding an internal agreement factor *R*_int_ = 0.093.

The intensity distribution across reflection orders is unusual, with main reflections exhibiting intensities comparable to those of first-order satellite reflections (see also Section 4). In most modulated structures, satellite reflections are typically significantly weaker than the main reflections, as they arise from a relatively small periodic distortion of an average structure. In contrast, the comparable intensity observed here indicates a strong structural modulation, making the structure solution particularly challenging within the superspace formalism.

The appropriate superspace group was identified as *P*1(σ_1_σ_2_σ_3_). Two alternative structure-solution strategies were initially considered: (1) a direct structure solution in superspace using the charge-flipping algorithm (as successfully applied for compound **1**), and (2) a two-step strategy consisting of solving the average structure first, followed by modeling the modulation.

The first approach was unsuccessful, most likely because the strength and complexity of the modulation exceeded the capability of the single-harmonic description assumed in the *Superflip* implementation. The second approach, involving the solution of the average structure using only the main reflections, also failed when tested with several commonly used programs [*SHELXT*, *SHELXS* (Sheldrick, 2008 ▸); *SHELXD* (Schneider & Sheldrick, 2002 ▸); *Olex2.solve* (Bourhis *et al.*, 2015 ▸)].

A similar situation has been reported previously (Schönleber & Chapuis, 2004 ▸), where information from a commensurate superstructure approximant was used to obtain starting values for both atomic coordinates and modulation parameters. In the present study, an alternative strategy implemented in *JANA2006* (Petříček *et al.*, 2014 ▸) was adopted. This method consists in generating an artificial dataset in which the intensity of each main reflection is enhanced by summing it with the intensities of its associated satellite reflections. The resulting dataset effectively represents a reinforced approximation of the average structure.

This modified dataset was successfully processed using *SIR2019* (Burla *et al.*, 2015 ▸) yielding ultimately a stable solution for the average structure. Twelve carbon atoms, two nitro­gen atoms, and three oxygen atoms were identified in the electron-density map. Subsequently, twenty-six hydrogen atoms were introduced based on geometrical considerations using *JANA2020* (Petříček *et al.*, 2023 ▸). During refinement, both atomic displacement parameter (ADP) restraints and geometrical restraints were applied to the hydrogen atoms.

Refinement of the average structure with isotropic ADPs yielded an *R* factor of approximately 55% for the main reflections and, as expected, 100% for the satellite reflections. These high residual values clearly demonstrate that the average structure alone is insufficient to describe the diffraction data of such a strongly modulated system.

An additional difficulty in the refinement arises from the chemical composition of the compound, which contains only light atoms (C, N, O and H) and no heavy atoms with high scattering power. In many modulated structures containing heavy elements, the modulation can initially be modeled by refining the displacement parameters of the dominant scatterers, which often provides sufficient phase information to describe the satellite reflections and stabilize the refinement. In the present case, however, no single atom type contributes disproportionately to the scattering, and the modulation is distributed over the entire molecular framework.

As a consequence, it was not possible to obtain a stable description of the modulation by refining the displacement of a limited subset of atoms. Instead, the refinement had to be carried out in a collective and progressive manner, involving all atoms in the structure from the early stages of the superspace refinement. A first-order harmonic wave was initially applied, resulting in a modest but measurable improvement of the model. Higher-order harmonic components were then introduced sequentially, in accordance with the experimental observation of satellite reflections up to the fourth order. After each refinement cycle, the model quality improved significantly, confirming the physical relevance of the additional modulation terms.

To further stabilize the refinement, the damping factor controlling the least-squares parameter shifts was deliberately reduced during the initial refinement cycles, thereby preventing divergence and ensuring gradual convergence of the model.

In the final stages of refinement, anisotropic atomic displacement parameters were introduced for all non-hydrogen atoms. At the same time, the modulation model was simplified to retain only the statistically significant harmonic components. All modulation parameters with values smaller than three times their standard uncertainty were fixed to zero in order to avoid over-parameterization and to ensure model stability.

The final refinement involved 657 refined parameters. For comparison, the previously published supercell model required 1378 parameters for 10918 observed reflections and yielded an agreement factor of 11.62%. In contrast, the present superspace approach provides a significantly improved description of the diffraction data while using fewer refined parameters, demonstrating both the physical validity and the efficiency of the modulated structure model.

Furthermore, a substantial reduction in the refined ADPs is observed in the present model compared to those in the initial supercell approximant. This observation strongly suggests that the abnormally large displacement parameters reported in the original supercell description were most likely artifacts arising from the use of a supercell approximation.

### 3.3. Structure solution and refinement of **3**

Crystals of **3** were obtained in an attempt to grow crystals of **1** from methano­lic solutions. Only after the structure solution it appeared that a symmetrized octyl-ureido structure had been serendipitously obtained without any hy­droxy group. No sign of twinning or modulation was detected in the reconstructed diffraction images. Structure solution and refinement proceeded smoothly without any difficulties.

## 4. Modulation strength in the structures of **1** and **2**

Based on the visual inspection of the reconstructed diffraction images in selected planes (Figs. 2 ▸ and 3 ▸) it seems that the modulation in both phases is particularly strong, but there is there is currently no generally accepted definition of what should be considered as ‘strong’. Three different approaches can be used to quantify the strength of the modulation, one in real-space and two other related ones in reciprocal space. The real-space approach considers the modulation amplitudes, either in absolute length units or in relative units with respect to the lengths of the cell axes. While this seems appealing, it considers the amplitudes of different atoms of different nature and it is not clear how to obtain one overall modulation strength parameter for the whole structure. A reciprocal space approach based on the diffraction intensities has the advantage that an overall modulation strength parameter which represents the whole structure is automatically obtained. The intensity statistics of the main and satellite reflections give a first indication of the modulation strength. Table 2 ▸ gives the statistics for the structures **1** and **2**.

Whereas the mean intensity of first-order satellites 〈*I*/σ(*I*)〉 [*I* > 2σ(*I*)] for **1** is 80.4% of that for the main reflections, for **2** first-order satellites are on the average nearly as strong as the main reflections (98.8%). However, this still does not give one overall parameter that characterizes the strength of the modulation. We therefore calculated the percentage of the total diffraction intensity that is distributed in the satellite reflections, which amounts to 54% for **1** and 67% for **2**.

**Figure 5:**
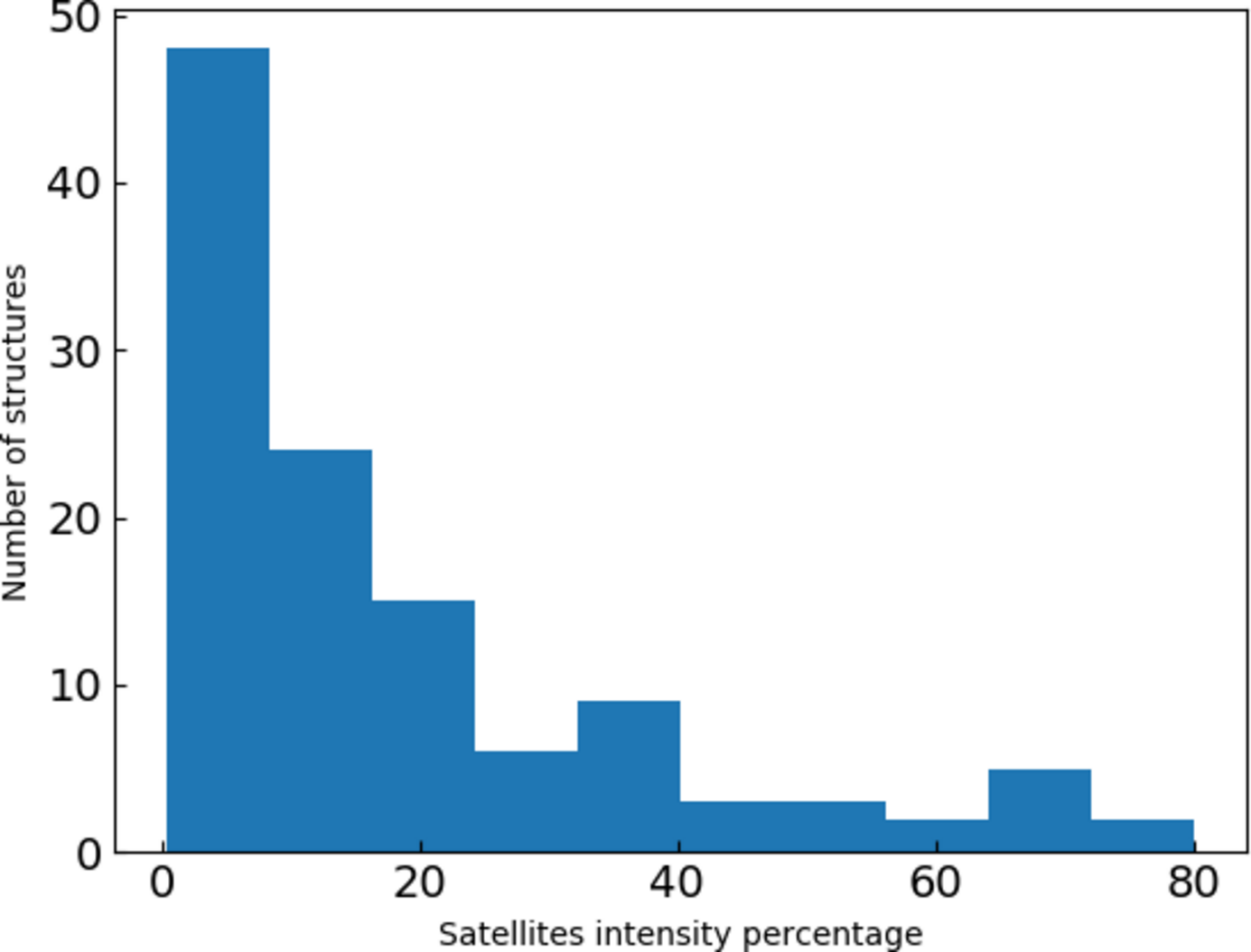
Histogram of the diffraction intensity that is scattered in the satellite reflections for 117 different modulated structures.

It is tempting to compare these numbers to those obtained for other modulated structures. We analyzed the observed intensity statistics for 117 modulated structures, irrespective of their chemical nature (hybrid: 2, inorganic: 85, metal–organic: 14, organic: 16) or the radiation used to collect the data (X-rays, neutrons, electrons), the method (single-crystal or powder), single domain or twinned, the modulation dimension (1 or 2) or the method used (single-crystal or powder). Some datasets from strictly commensurately modulated structures with low denominators in their *q* values were excluded, because they could give rise to particular unusual features such as the mean intensity of the seventh-order satellites being equal to that of the main reflections and virtually no intensity for the other satellite orders (Pavlyuk *et al.*, 2014 ▸). Fig. 5 ▸ gives a histogram of the percentage of the total intensity that is distributed in the satellites (Γ_sat_). The 50% percentile of the distribution, *i.e.* the value of under which 50% of the observed values fall (median), is calculated to be 11.4%, whereas the 90% percentile is 49.0%, *i.e.* 10% of the observed intensity distributions of modulated structures has a Γ_sat_ value larger than 49.0%. Based on the (arbitrarily chosen) 50% and 90% percentile values we can consider the strength of the modulation being normal if it is below Γ_sat_ = 11%, strong if 11% < Γ_sat_ < 49%, and very strong if Γ_sat_ > 49%. Both **1** (Γ_sat_ = 54%) and **2** (Γ_sat_ = 67%) have modulations that can be considered as very strong. The incommensurately modulated molecular structure of C_6_H_4_S_2_AsCl which was reported to have strong satellite intensities falls also in this category with Γ_sat_ = 60%.

In many inorganic materials, modulated phases are observed during phase transitions leading to states with distinct electronic properties such as charge-density waves, ferroelectricity, or magnetism. The non-modulated ground state defines the average structure of the material, which becomes periodically perturbed in the modulated phase. This perturbation results in weak or moderate modulations according to our classification.

However, certain inorganic crystals display very strong modulations that originate from their intrinsic structural arrangements. This is the case for the [Bi_2_Sr_3_Fe_2_O_9_]m[Bi_4_Sr_6_Fe_2_O_1__6_] family, where the observed diffraction patterns closely resemble those reported for compound (2), notably exhibiting waves of intense reflections alternating with regions of lower intensity [see Fig. 4 ▸ in Elcoro *et al.* (2012 ▸)].

The observation of diffraction planes presenting a high density of diffraction spots with wave-like modulations in diffracted intensity appears to be a distinctive hallmark of these highly modulated structures, and may guide the crystallographer toward an interpretation in terms of incommensurately modulated order.

Other structures have been reported to have very strong incommensurate modulations, such as the tungsten bronze-type structure of PbBiNb_5_O_15_ (Lin *et al.*, 2015 ▸). From a closer look at the published details it appears that the characterization as ‘unusual’ by the authors originates from satellite reflections observed up to the fifth order in selected-area electron diffraction images, whereas in the X-ray powder diffraction data only first-order satellites have been observed which amount to only 3% of the total diffraction intensity. Electron diffraction can indeed be every effective in observing high-order satellites (Li *et al.*, 2020 ▸), but it does not necessarily mean that the modulation is strong.

The benchmark set of modulated structures used for the calculation of Γ_sat_ can also be used to verify whether the mean intensity of the satellites decreases exponentially with the reflection order, which is sometimes put forward as the usual behavior of the intensity decrease of satellites of modulated structures (Rekis *et al.*, 2021 ▸). This is approximately true although the data for higher reflection orders are evidently rather scarce. It can also be seen that in some cases the mean intensity of a reflection order is higher than that of the main reflections.

The supporting information gives all information and detailed statistical information, as well as the sources – in the form of the digital object identifiers of the original scientific publication – of the 117 incommensurate structures used for this study. The reflection data have been extracted from different sources, in particular from the Bilbao Incommensurate Structures Database (Gabirondo-López *et al.*, 2024 ▸), but also from the electronic supplementary information archives of different journals. The script to produce the statistics of Figs. 5 ▸ and 6 ▸ is also included.

## 5. Description and origin of the modulated structures

### 5.1. General packing features

Both structures **1** and **2** consist of molecular double layers packed along the *c* axis (Figs. 7 ▸ and 8 ▸). The two individual layers within each sandwich are hold together by strong intermolecular HO⋯HO hydrogen bonds. Within each molecular layer the molecules bind through strong NH⋯O hydrogen bond urea–urea interactions in the *b* direction for **1** and in the *a* direction for **2**. The urea–urea interactions also determine the cell repeat distances in these directions, *i.e.* 4.619 (1) Å and 4.6380 (4) Å for **1** and **2**, respectively, *i.e.* the distance between two urea groups. The double layers interact to each other by weak van der Waals interactions between the aliphatic methyl end groups.

### 5.2. The modulated structure of **1**

Structural modulations can be described in different ways, either by tracing individual atom displacements with respect to their position in the basic unit cell, or by tracing selected geometric angles and torsion angles versus the modulation parameter *t* (see also Section 8). The first way gives insight in the strength of the modulation, whereas the second way presents more insight in which parts of the molecule are affected. Fig. 9 ▸ gives the individual atom displacements as a function of *t*. The largest amplitudes are for the carbon atoms at the end of the aliphatic chain; the C1 position is modulated between 0.08 Å and 0.93 Å with respect to the average position, whereas the carbon positions not far from the urea motif have a much smaller modulation amplitude. The displacement amplitudes never reach zero amplitude, since the values are composed of phase-shifted *x*, *y* and *z* individual vector components, which individually may reach and cross the zero line, but in most cases not at the same *t* value.

Fig. 10 ▸ shows the distribution of the bond-valence distance variations as a function of the modulation parameter *t*. The C=O distance of the urea motif shows the largest variations between 1.214 and 1.254 Å which is not exceptional in view of the observed C=O distances in similar urea motifs in the Cambridge Structural Database [514 observations 1.242 Å with a standard deviation of 0.018 Å; CSD version 6.00; Groom *et al.* (2016 ▸)]. Other intramolecular bond distances show very little variation along the modulation.

The largest intramolecular modulations occur in the rotatable bonds around the pivot between the alkyl chain and the urea group (Fig. 11 ▸), whereas the rotatable bonds within the urea group are also involved. In both cases all torsion angles fall within the limits of observed values for similar structural fragments (Mogul; (Bruno *et al.*, 2004 ▸)). It appears that the modulations of the C6—C7—N1—C9 and C7—N1—C9—N2 torsion angles which connect the alkyl chain with the urea motif are in phase but out of phase with the N1—C9—N2—C10 torsion angle which connects the urea group to the 1,3-di­hydroxy­propan-2-yl group.

The (9*a*,*b*,2*c*) supercell approximant of the basic unit cell gives another pictorial representation of the structural modulations in **1** (Fig. 12 ▸).

The modulation wave is clearly visible within the hy­droxy channel, whereas the interface between adjacent terminal alkyl methyl group is hardly affected. Interestingly the pair of intermolecular O–O hy­droxy hydrogen bond distances is hardly affected as well despite the visual modulation, whereas the pair of intermolecular N–O urea hydrogen bond distances is strongly modulated (Fig. 13 ▸)

### 5.3. The modulated structure of **2**

Fig. 14 ▸ presents the atomic displacements in the structure of **2** with respect to their position in the basic cell. The displacements are in general somewhat larger than in the structure of **1**, but the main pattern is observed, *i.e.* the carbon atoms at the unconnected end of the aliphatic chain have the largest displacements and amplitudes, whereas the carbon atoms close to the urea group have relatively small amplitudes and medium displacements. The oxygen atoms of the two hy­droxy groups have on the other hand large modulation amplitudes and strong displacements, but they are nearly in phase with the terminal carbon atoms of the octane chain, whereas they are out of phase in the structure of **1**.

The bond distance modulations (Fig. 15 ▸), the torsion angles (Fig. 16 ▸) and the intermolecular hydrogen bond modulations (Fig. 17 ▸) follow also the trend of those in the structure of **1**, but the intermolecular hy­droxy hydrogen-bonds are stronger modulated in the structure of **2** than in the structure of **1**.

Fig. 18 ▸ gives a projection along *a* of the approximant (*a*, 9*b*, 7*c*) supercell for **2** providing a global overview of the effect of the modulation.

### 5.4. The non-modulated structure of **3**

Compound **3** was obtained when trying to synthesize **1**. The urea group is central to two octyl chain groups so that the obvious supramolecular assembly only takes place via urea–urea interactions. These interactions again dictate one of the cell axes being equal to the urea–urea distance [*b* = 4.5897 (4) Å, Fig. 19 ▸(*a*)] whereas the *c* axis [16.146 (2) Å, Fig. 19 ▸(*b*)] is equal to the repeat distance between stacked single molecular layers, in between which only weak van der Waals type interactions play a role. Within the molecular layers no short intermolecular interactions are found in the direction perpendicular to the linear urea chains. The absence of the hy­droxy–hy­droxy hydrogen bonds orthogonal to the urea–urea hydrogen bonds in **3** may explain the absence of structural modulations in this structure, but other similar structures have been reported with hy­droxy–hy­droxy groups concomitant with orthogonal urea–urea interactions without structural modulation (Huang *et al.*, 2021 ▸). The data sets from that publication have been rechecked for the presence of overlooked satellite reflections, but this appeared not to be the case.

## 6. Origin of the structural modulation

Several theories have been proposed to explain the structural modulations observed in crystalline molecular organic compounds. These theories have been developed in the context of both commensurately and incommensurately modulated structures (Schoenleber, 2011 ▸). They also help interpret the occurrence of high-*Z*′ structures (Steed & Steed, 2015 ▸; Johnstone *et al.*, 2010 ▸; Brock, 2016 ▸) which are frequently identified as (in)commensurately modulated but are typically refined using a supercell approximant. The prevailing perspective suggests that competition between different interactions can lead to frustration, resulting in structural modulations. It is important to distinguish between structurally flexible molecules—those with one or more torsional degrees of freedom—and rigid molecules. In flexible molecules, the interplay between conformational variability (intramolecular energy) and packing features driven by intermolecular interactions, such as hydrogen bonds (Canadillas-Delgado *et al.*, 2019 ▸) or weaker van der Waals interactions (Dzyabchenko & Scheraga, 2004 ▸), can promote frustration and, consequently, structural modulation. Additionally, competition between different intermolecular forces, such as less-directional van der Waals interactions and more-directional hydrogen bonds, has been identified as a cause of structural frustration, leading to modulation in the form of high-*Z*′ structures (Anderson *et al.*, 2008 ▸). For rigid molecules, such as those found in urea inclusion compounds, a mismatch in size between guest and host molecules can create packing frustration, resulting in structural modulation. Another proposed explanation for structural modulation is the optimization of hydrogen-bond interactions to minimize total lattice energy (Rekis *et al.*, 2021 ▸).

For the two modulated hy­droxy channel structures **1** and **2** of this study the urea–urea interactions direct the packing in the non-modulated direction (*b* for **1** and *a* for **2**) between neighboring alkyl chains. In both structures the alkyl packing is hardly modulated along the modulation vector and the modulation is concentrated in the hy­droxy channels. The hy­droxy hydrogen bond distances through the channels themselves are only weakly modulated, but instead the center of masses of the entire (1,3-di­hydroxy­propan-2-yl) modulate smoothly through the 3D structure, keeping the hydrogen bond distances approximately equal. The existence of two strong competing perpendicular hydrogen bond interactions, *i.e.* the urea–urea ones and the hy­droxy-hy­droxy ones, could lead to a relaxation of the latter through a structural modulation leading to a lower-energy structure. This cannot be more than a hypothesis, since, as outlined earlier, similar compounds having both urea–urea interactions and hy­droxy channels do not lead to modulated structures.

## 7. DFT calculations

To gain deeper insight into the energy variations along the modulation parameter *t* in the structures of compounds **1** and **2**, density functional theory (DFT) calculations using the *Gaussian16* software package (Frisch *et al.*, 2016 ▸) were performed. It should be noted that these energy variations are only related to the amount of conformational strain along *t*, but that they do not explain the origin of the modulation. Structural snapshots were exported from *JANA2020* at 20 equidistant *t* values between 0.00 and 0.95. C—H atomic distances were renormalized to 1.089 Å, N—H distances to 1.015 Å, and O—H distances 0.993 Å. Single-point energy calculations were performed using both M062X (54% Hartree–Fock exchange) (Zhao & Truhlar, 2008 ▸) and B3LYP (20% Hartree–Fock exchange) (Becke, 1993 ▸; Lee *et al.*, 1988 ▸) hybrid functional in conjunction with the def2-TZVP basis set (Weigend & Ahlrichs, 2005 ▸). As shown in Fig. 20 ▸, both hybrid functionals yielded highly consistent results, with the relative energy varying clearly as a function of the modulation parameter *t*.

For compound **1**, only a modest energy fluctuation (±7 kJ mol^−1^) was observed along the modulation coordinate [Fig. 20 ▸(*a*)]. This variation arises from a subtle yet meaningful bending of the hy­droxy-ureido moiety anchored to the 2-hexyl alkyl chain [Fig. 20 ▸(*b*)], driven by the intramolecular hydrogen-bonding network discussed above (see Fig. 12 ▸). In contrast, the linear compound **2** exhibits a much larger energy modulation amplitude [∼90 kJ mol^−1^, Fig. 20 ▸(*c*)], corresponding to a pronounced bending of the alkyl tail [Fig. 20 ▸(*d*)]. To better quantify this deformation, the deviation from linearity was defined as the difference between 180° and the calculated bending angle. As shown in Fig. 20 ▸, a strong correlation emerges between this deviation and the DFT-computed relative energies, with the maximum energy amplitude coinciding with the largest bending deviation (∼8°). The energy variances for both **1** and **2** along *t* are considerably larger than those determined for the saccharinate anions in the modulated structure of sodium sacchiranate 1.875·hydrate (∼3 kJ mol^−1^), the only other case reported for which similar single-point energy calculations have been performed (Rekis *et al.*, 2020 ▸).

It is worth emphasizing that packing interactions in the solid state compensate for such energy increases. Indeed, the solid-state structure is stabilized by multiple non-bonding interactions absent in the gas phase, leading to energy differences between experimental solid-state geometries and the ideal gas phase global minima. For compound **1**, the optimized gas-phase geometry at the M06-2X/def2-TZVP level is approximately 47 kJ mol^−1^ more stable than the average solid-state conformation, primarily due to relaxation of the urea moiety, which in the gaseous phase is not engaged in the directional hydrogen-bonding network observed in the solid state. Similarly, compound **2** displays an even greater stabilization (∼150 kJ mol^−1^) upon optimization, reflecting relaxation of both the alkyl chain (elimination of bending) and the ureido group, as well as the C—O—H angle adjustment in the absence of the hydrogen-bonding channel network present in the crystal structure.

## 8. Visualization of modulations by 3D Lissajous curves

In modulated structures, such as incommensurate crystals, the atomic positions can be described as periodic functions of the internal coordinate *x*_4_. The displacement *u*^*j*^ of each atom is therefore represented by a linear combination of sinusoidal functions, corresponding to the Fourier series expansion of the modulation wave developed up to the *n*^th^ order:
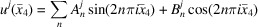
Modulations are typically visualized in so-called *t*-plots (as in Section 5), where geometrical parameters such as inter and intramolecular distances, angles and torsions are traced as a function of the modulation period *t*. This parameter is defined as *t* = *x*_4_ − **q**·(**L **+ **r**_0_) with **L** the lattice vector, *q* the modulation vector and **r**_0_ the average position. All geometrical parameters can then be obtained by computing the atomic positions **r** = **r**_0_ + **u**(*t* + **q**·**r**_0_) as a function of *t*.

Although this gives the most precise information about the structural consequences of the modulation for a certain geometrical parameter, a more visual representation of the modulation is often desirable for the structure as a whole. Programs such as *Jmol* (Hanson, 2010 ▸) or *JANA2020* (Petříček *et al.*, 2023 ▸) offer such an option, generating animations in which each atom ‘vibrates’ as a function of *t*, with *t* interpreted as time. This is the current preferred way for the representation of modulated structures in *Acta CrystallographicaSection B* based on the definitions in the modulated and composite structures dictionary (msCIF, https://www.iucr.org/__data/iucr/cifdic_html/1/cif_ms.dic/index.html).

Here, we present an alternative way to visualize modulated structures, based on so-called Lissajous (also called Bowditch) curves (Lissajous, 1857 ▸). Lissajous curves correspond to the trajectories generated when several sinusoidal oscillations—potentially with different amplitudes and phase shifts—combine in a multidimensional space. They can be expressed as
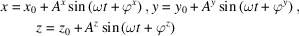
Lissajous curves therefore offer a natural geometric representation of harmonic displacements. When a modulation is purely harmonic, the atomic trajectories in three-dimensional space are, by definition, Lissajous curves. If the modulation contains higher Fourier terms (*n* > 1)(*n* > 1)(*n* > 1), the trajectory is no longer a simple elementary Lissajous curve but a superposition of multiple such curves. The resulting figure becomes more intricate, yet remains fundamentally of the same nature—a combination of periodic sinusoidal components forming quasi-Lissajous trajectories.

Visualizing atomic modulations in terms of Lissajous curves provides several advantages. It allows one to clearly depict the spatial shape of the modulation (elliptical, circular, or more complex), to visualize the relative phase relationships between its components, and to obtain an intuitive geometric representation of the harmonic periodicity inherent in the modulation function. Moreover, this approach establishes a direct and visually transparent link between the geometrical characteristics of the modulation and the Fourier coefficients that define the crystallographic model. Conveniently, software capable of generating such structural Lissajous figures already exists, most notably *MoleCoolQt* (Hübschle & Dittrich, 2011 ▸), even if it was not originally designed for this purpose.

Fig. 21 ▸ gives the Lissajous representation of the modulation of the structures **1** and **2**. They give immediately an insight in the strength of the modulations, *i.e.* it is easily seen that most of the individual displacements have only a slightly lower magnitude as the typical covalent bond distance in this molecular structure. The color scheme along each Lissajous curve represents the progression along the modulation parameter *t*.

Whilst the global representation of structural modulations using Lissajous curves is not more than an alternative to the *Jmol* representation (Hanson, 2010 ▸), it has the advantage that it does not give the impression of vibrating atoms. The color scheme along the curves immediately permits to see which displacements are in phase or out of phase and they give also a very clear impression of the magnitudes of the displacements which are nearly as large as the covalent interatomic distances.

## 9. Discussion and conclusions

This study presents the results of a detailed reciprocal and real space examination of unusually strong modulations in two related organic hy­droxy channel structures. Whereas the diffraction pattern of a structure with a modulation of ordinary strength is easily recognized as a basic set of main and relatively strong reflections decorated with another set of much weaker satellite reflections, the diffraction patterns of very strongly modulated structures are much less easily decomposed into a clear hierarchy of main and satellite reflections.

Certain crystal structures, despite producing strong diffraction patterns, are classified as ‘unsolvable’ because they cannot be indexed using automated methods. This challenge mirrors the difficulties encountered with heavily twinned structures, which also may resist indexing when standard automated procedures fail. In the case of twinned structures, however, progress has been made through the development of semi-automated techniques that may decompose the patterns into those of individual twin domains.

For both twinned and modulated structures, successful indexing begins with a thorough examination of the unwarped diffraction planes—a step frequently overlooked due to overreliance on automated tools. Visual inspection of the intensity distribution in these planes should, however, prompt crystallographers to consider whether the pattern originates from a twinned or modulated structure.

In this study, the interpretation of the diffraction pattern for structure **2** was initially guided by the indexing of its superstructure approximant. Yet, this approach does not inherently lead to a clear decomposition into a basic reciprocal unit cell and modulation wavevectors, as the process of deriving these components from a reciprocal supercell lacks a unique solution. For structure **1**, not even a reciprocal superstructure unit cell could be reliably assigned, and no superstructure solution was achieved.

A shared feature in the diffraction patterns of both **1** and **2** is the presence of aligned ribbons of diffraction spots interspersed with reflection-free ribbons—a characteristic reminiscent of incommensurate diffraction patterns. Once recognized, the primary obstacle lied in distinguishing the main reflections from the first-order satellite reflections, which exhibit similar mean intensities. This distinction could only be resolved through iterative trial-and-error.

More broadly, this type of crystals may, following the failure of automatic indexing procedures, be prematurely dismissed as a poor-quality specimen and discarded. In such cases, the crystallographer returns to the center of the investigation and must, with an expert eye, regain control over the analysis of reciprocal space. The task is, in essence, to rediscover the underlying rules governing the diffraction pattern. These examples highlight the continuing need for specialized expertise at a time when single-crystal diffractometer manufacturers increasingly promote instruments as tools accessible to non-specialists. Far from rendering expertise obsolete, challenging diffraction patterns such as those described here demonstrate that human interpretation remains an essential component of crystallographic analysis.

The origin of the very strong modulations in these organic hy­droxy-channel structures remains unclear, although several hypotheses can be put forward. It is even not known whether ‘strong’ modulations, as characterized by, for example, the Γ_sat_ parameter, occur more frequently in inorganic than in organic structures, since the total number of organic structures in the reference data set is too small to draw firm statistical conclusions.

This relative lack of documented examples may itself contribute to the difficulty of recognizing and interpreting very strongly modulated diffraction patterns in molecular crystals. Historically, much of the methodological development and practical expertise in the analysis of modulated structures has emerged from studies of inorganic materials, where such phenomena are encountered more routinely. As a consequence, comparable cases in organic crystallography may be underreported, misinterpreted, or occasionally overlooked when automatic procedures fail, not because of a lack of competence, but rather because the relevant reference framework and accumulated experience are less extensive in this domain. The present study therefore illustrates the importance of maintaining and disseminating specialized crystallographic expertise across disciplinary boundaries.

There is a relation between structural modulations and reported high-*Z*′ structures, although not each high-*Z*′ structure is necessarily modulated, for instance structures with significantly different molecular conformations. Brock wrote in her study on high-*Z*′ structures that about 70% of high-*Z*′ organic structures include strong intermolecular interactions, (Brock, 2016 ▸) which is also the case for the 2 modulated structures of this study. This does not give in any way a clue why the latter modulations are particularly strong. A more systematic investigation would therefore be required to clarify the origin of very strong modulations in molecular crystals. In particular, a closer inspection of Brock’s database of 284 *Z*′ > 4 well determined organic structures and a superspace redetermination of those which can be categorized as (in)commensurately modulated, would be needed in order to shed light on the mechanisms underlying unusually strong structural modulations in incommensurate systems.

## Supplementary Material

Crystal structure: contains datablock(s) global, 1, 2, 3. DOI: 10.1107/S2052520626004932/dk5147sup1.cif

Intensity statistics (main and satellite reflections) of 117 modulated structures. DOI: 10.1107/S2052520626004932/dk5147sup2.pdf

**Figure 6:**
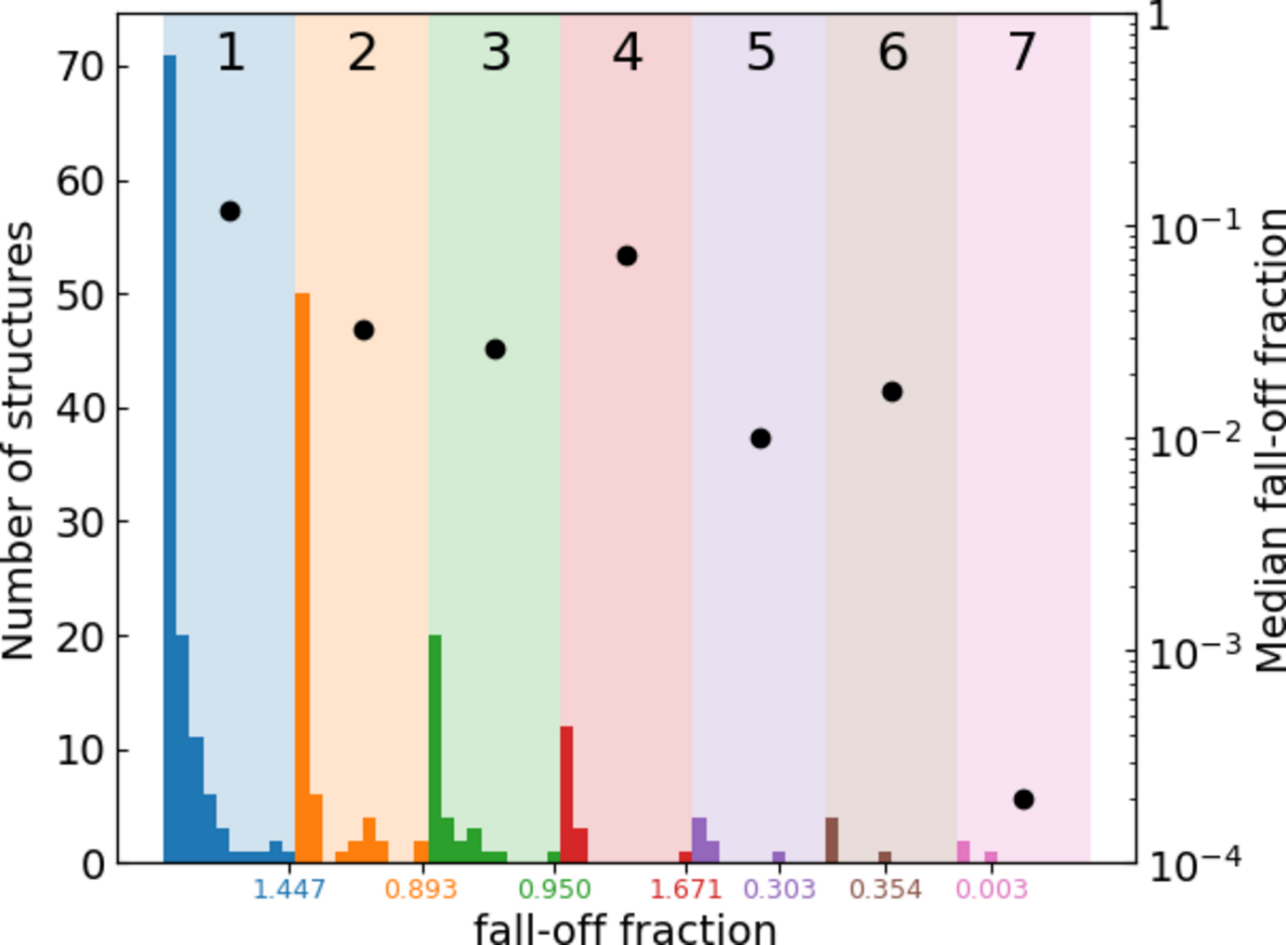
Histograms of the intensity fall-off fractions with respect to the intensity of the main reflections. The number on the horizontal axis represent the fall-off fraction of the last bin for each reflection order. The number of bins for each reflection order was taken as 10 if at least 10 structures were available and otherwise the number of structures available. The black dots are the median values of each histogram.

**Figure 7:**
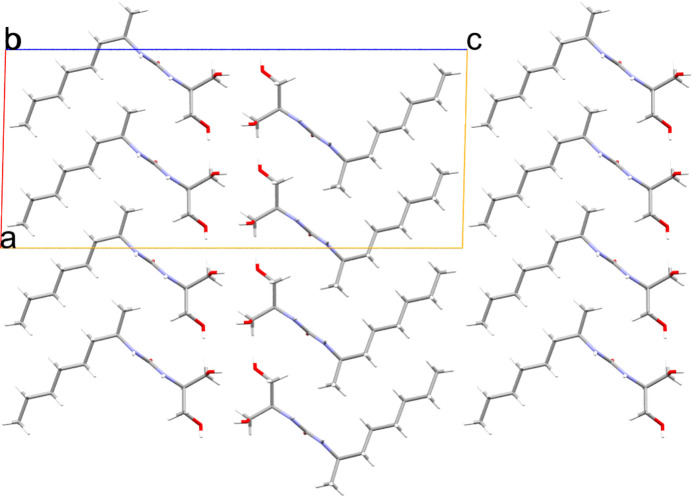
Packing of **1** along the ***b*** axis. The packing diagram has been drawn using the average structure.

**Figure 9:**
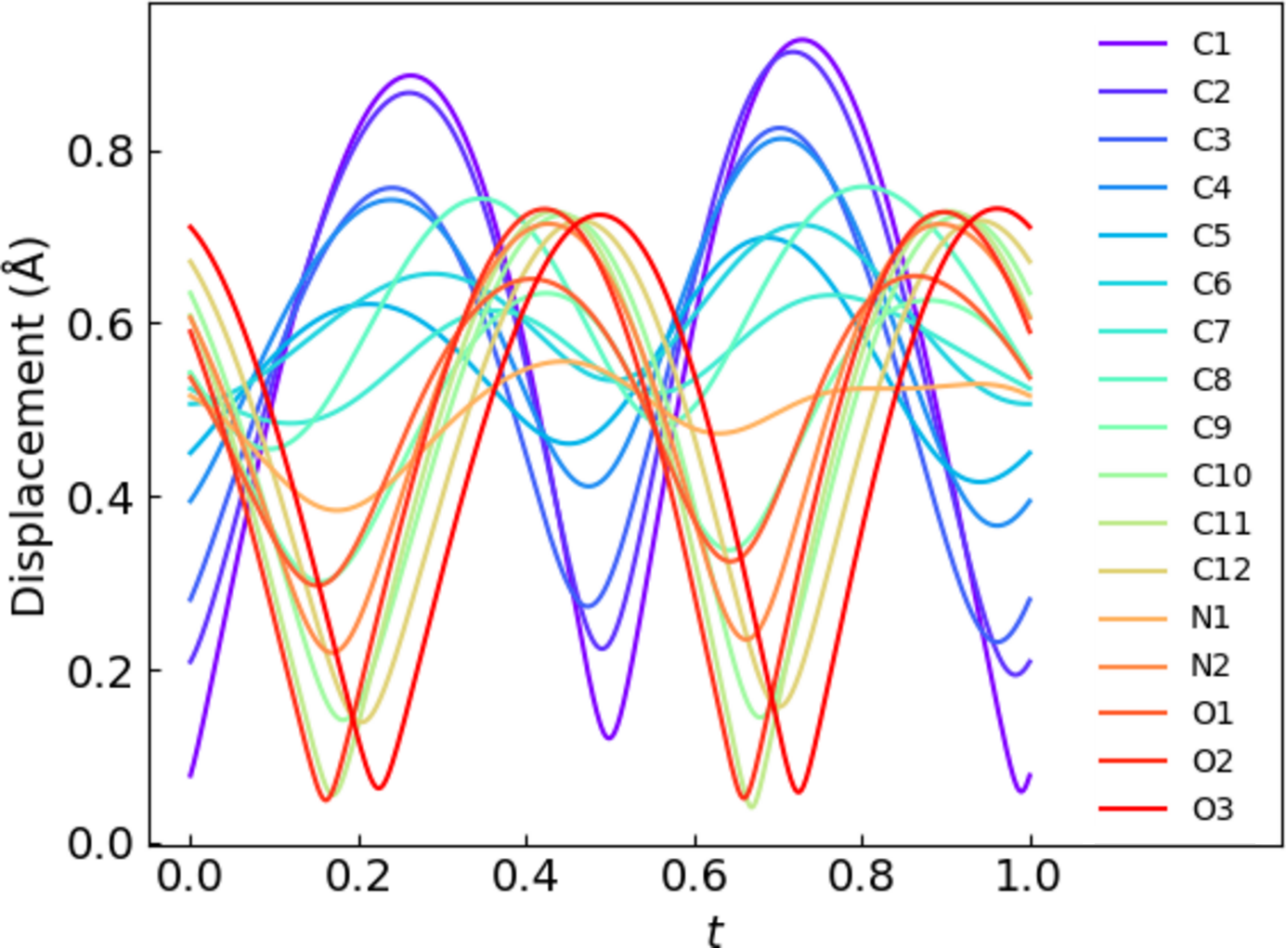
Displacements of the individual non-hydrogen atoms in the modulated structure of **1** with respect to their position in the average structure. For the atom labels, see scheme for **1** in Fig. 1 ▸.

**Figure 10:**
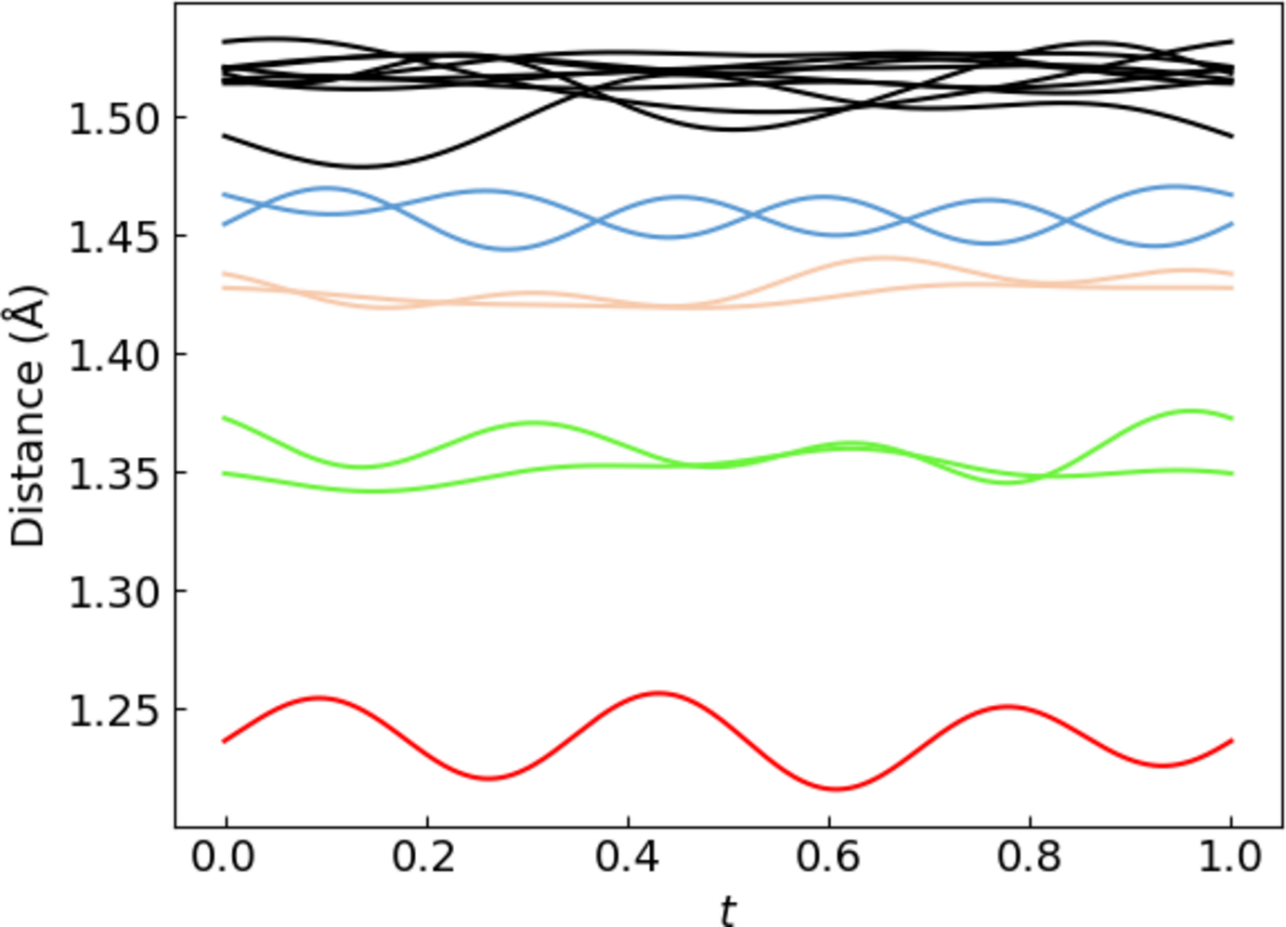
Covalent bond distance modulations as a function of *t* for structure **1**. The bond distances are color-coded according to the pictorial representation of **1** in Fig. 1 ▸.

**Figure 11:**
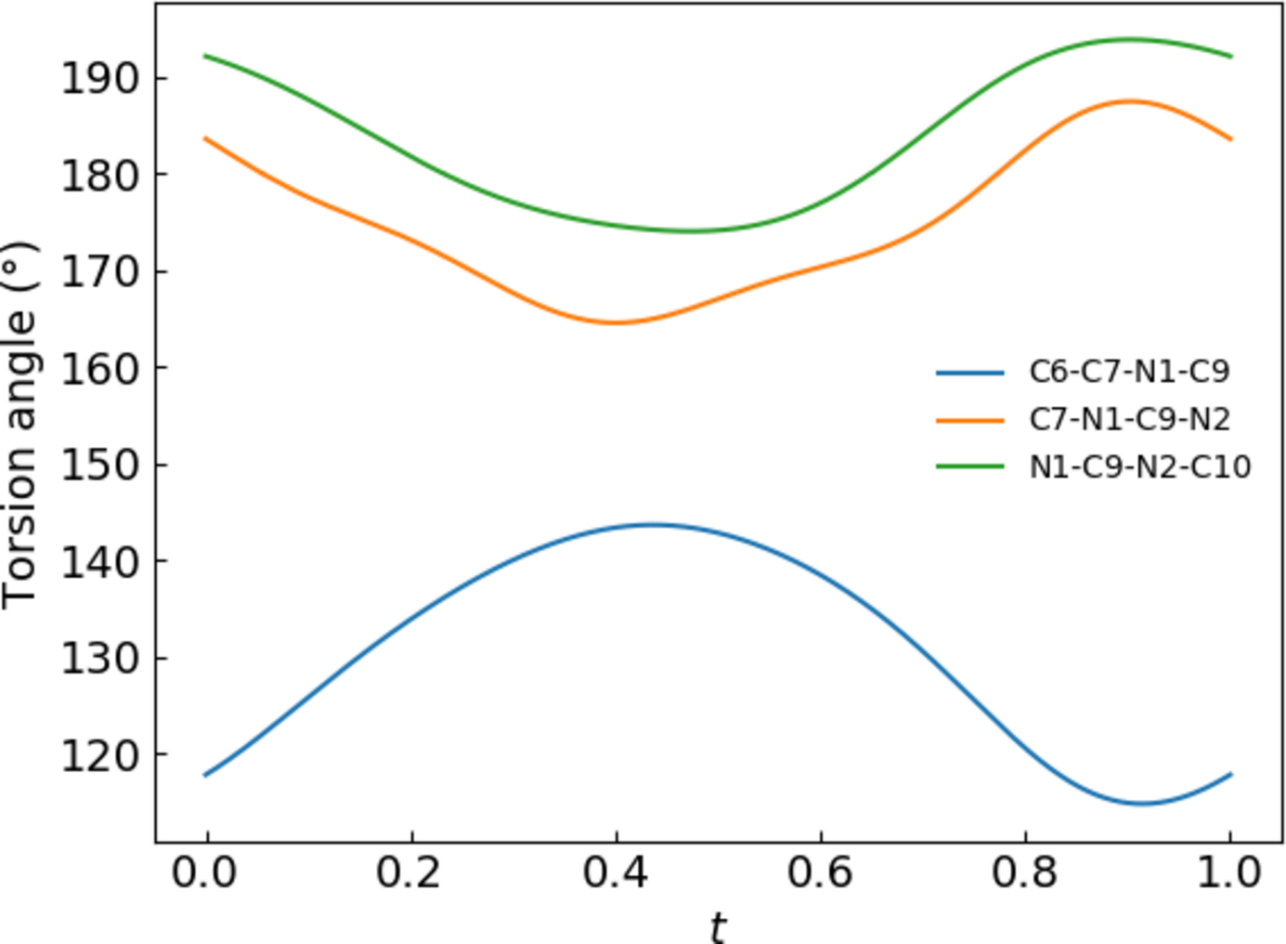
Selected torsion angles in the structure of **1** as a function of the modulation parameter *t*. For the atom labels, see scheme for **1** in Fig. 1 ▸.

**Figure 12:**
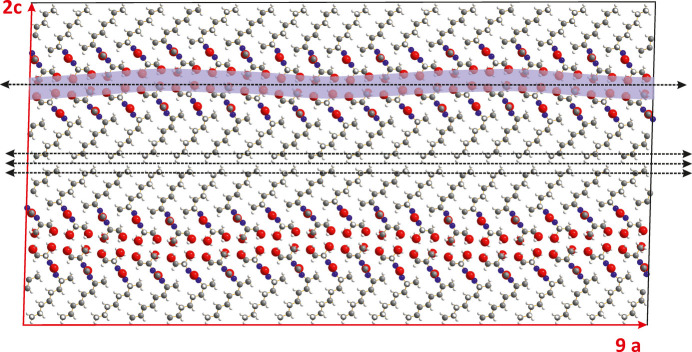
Projection along *b* of the (9*a*,*b*,2*c*) approximant unit cell of the structure of **1**. The purple ribbon enlightens the displacive modulation occurring at the level of the C-(COH)_2_ groups. The bidirectional arrows act as reference markers, illustrating the modulation effect: it is particularly pronounced in the area of the purple ribbon, while nearly negligible at the termini of the alkyl chains.

**Figure 13:**
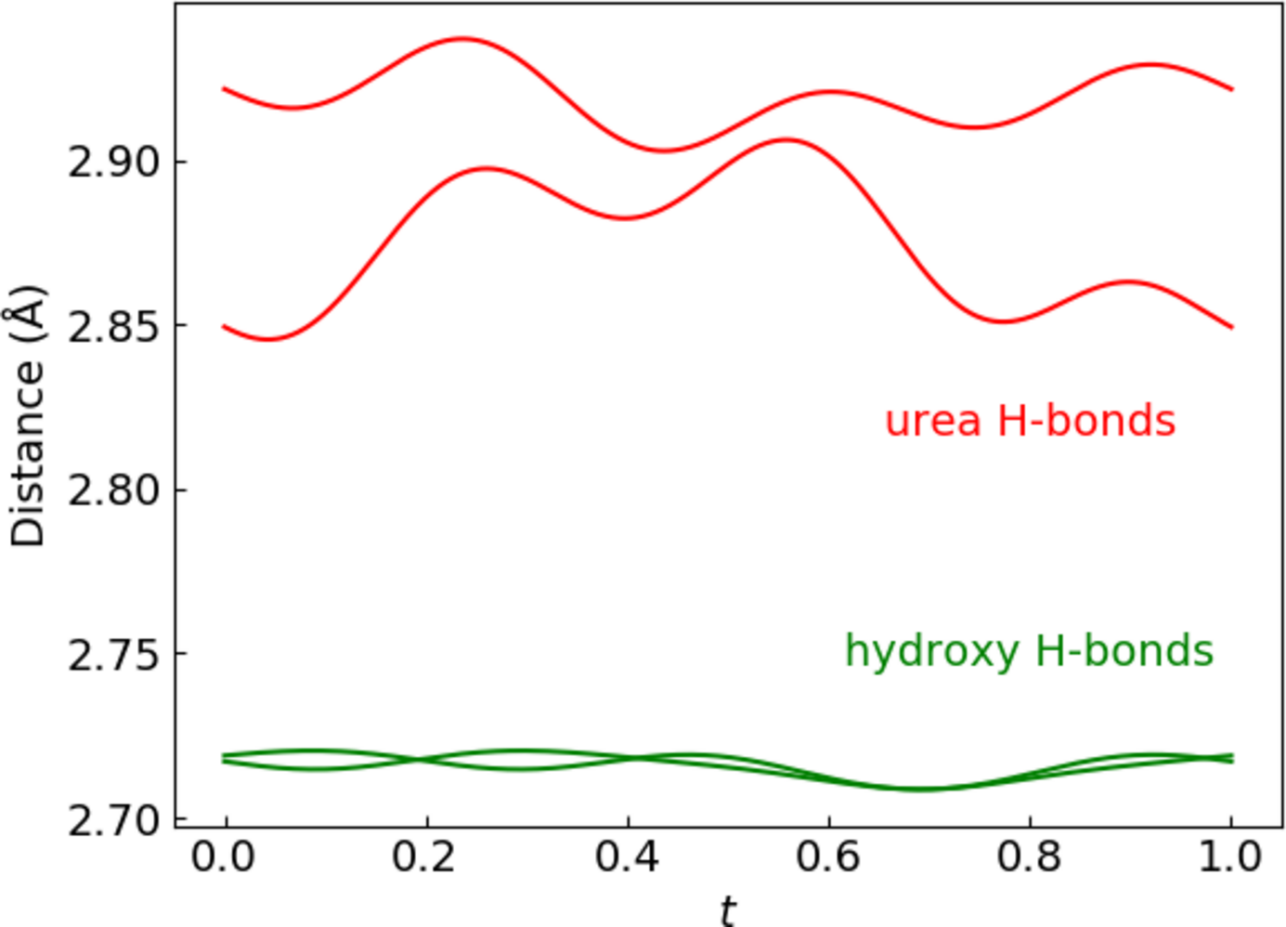
Intermolecular hydrogen bond distance modulations as a function of *t* in the structure of **1**.

**Figure 14:**
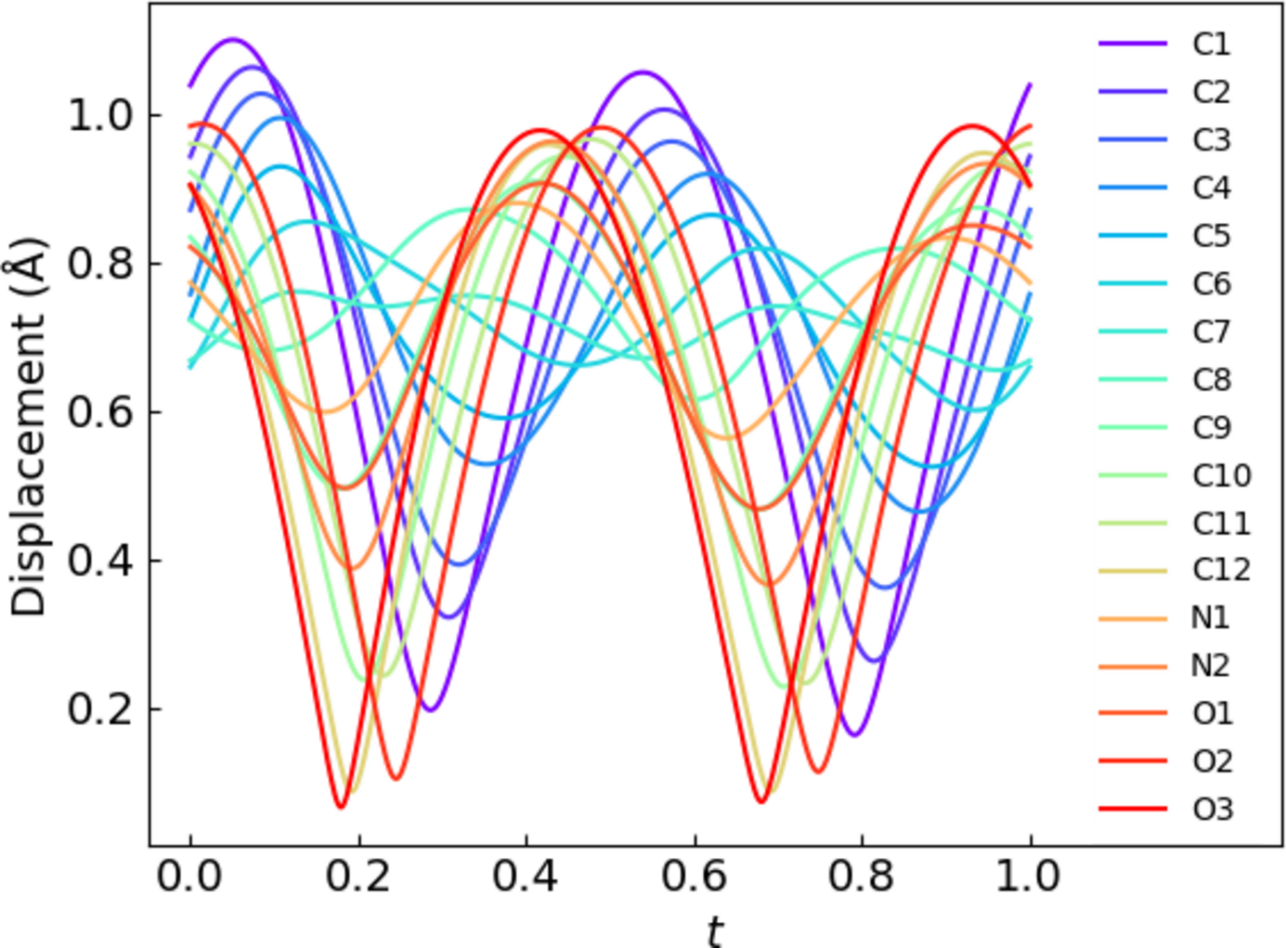
Displacements of the individual non-hydrogen atoms in the modulated structure of **2** with respect to their position in the average structure. For the atom labels, see scheme for **2** in Fig. 1 ▸.

**Figure 15:**
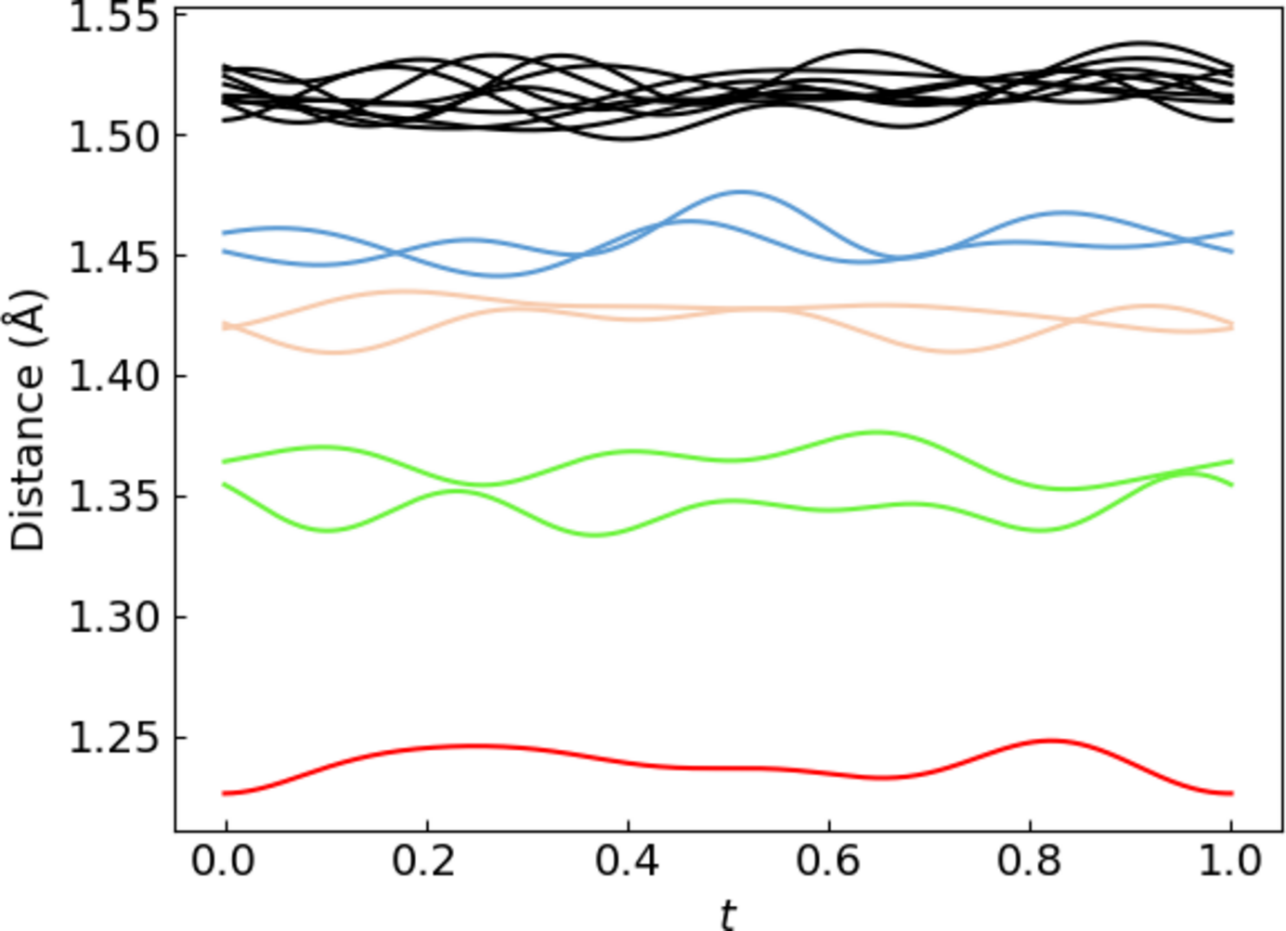
Covalent bond distance modulations as a function of *t* for structure **2**. The bond distances are color-coded according to the pictorial representation for **2** in Fig. 1 ▸.

**Figure 16:**
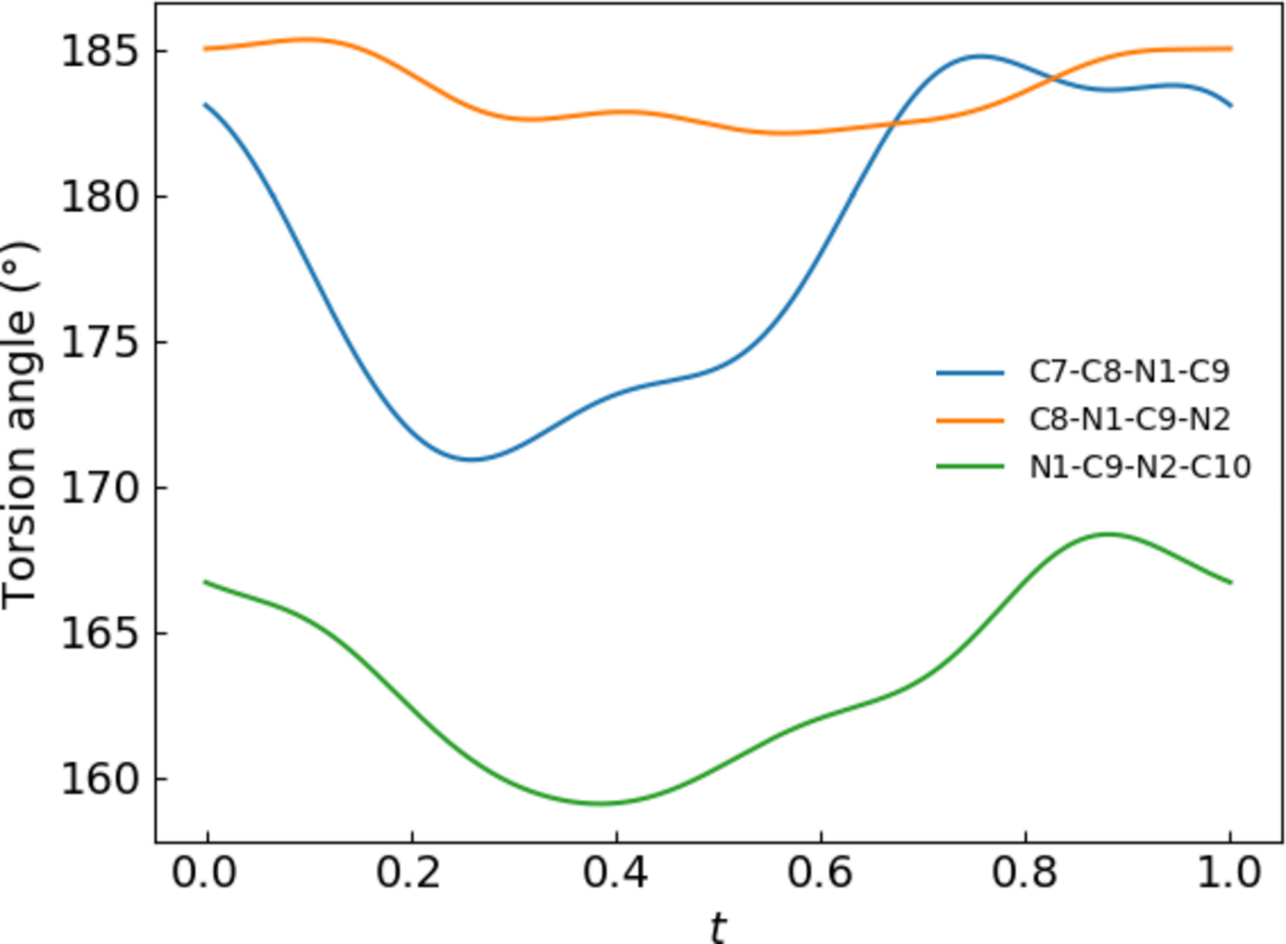
Selected torsion angles in the structure of **2** as a function of the modulation parameter *t*. For the atom labels, see scheme for **2** in Fig. 1 ▸.

**Figure 17:**
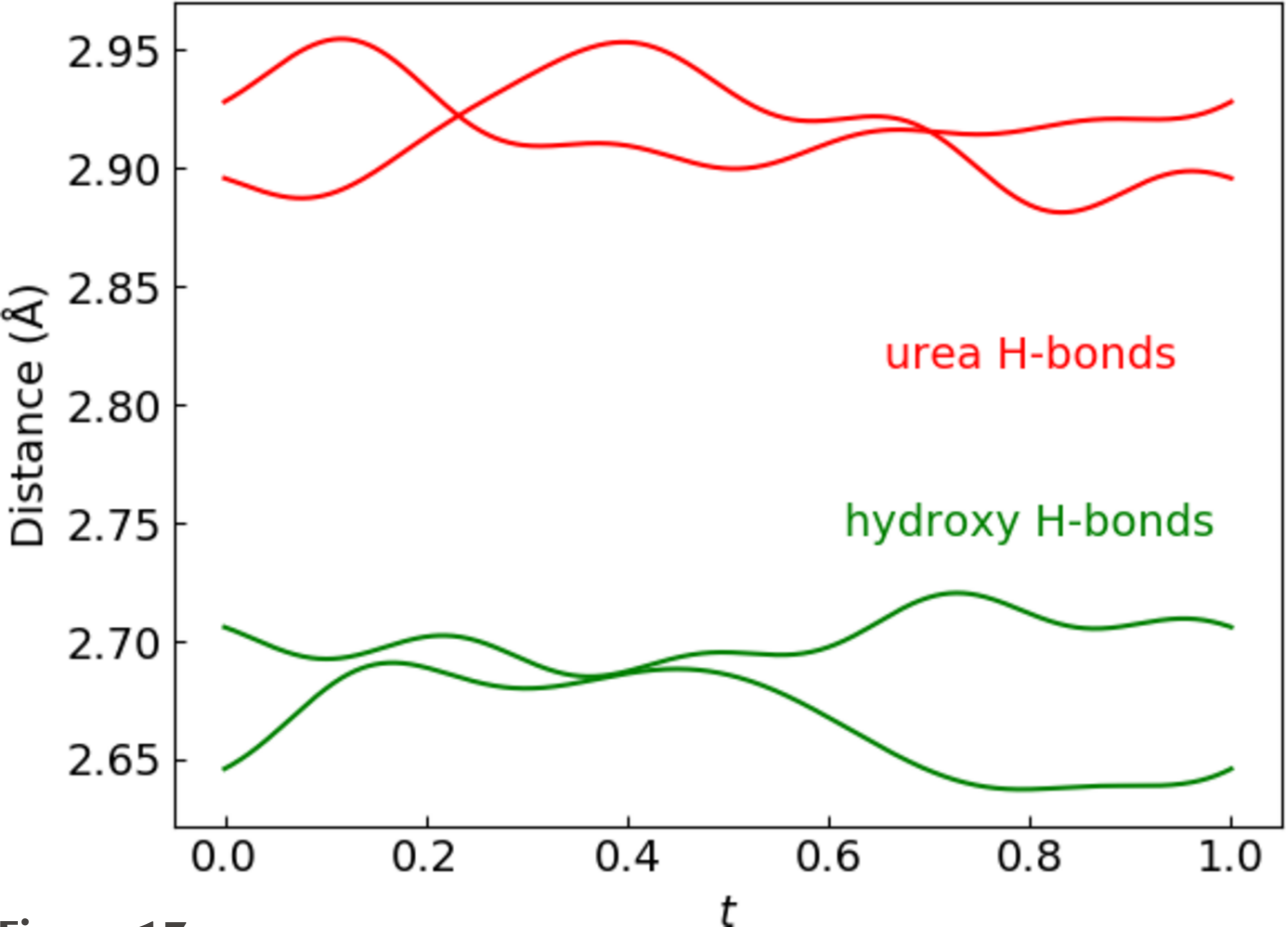
Intermolecular hydrogen bond distance modulations as a function of *t* in the structure of **2**.

**Figure 18:**
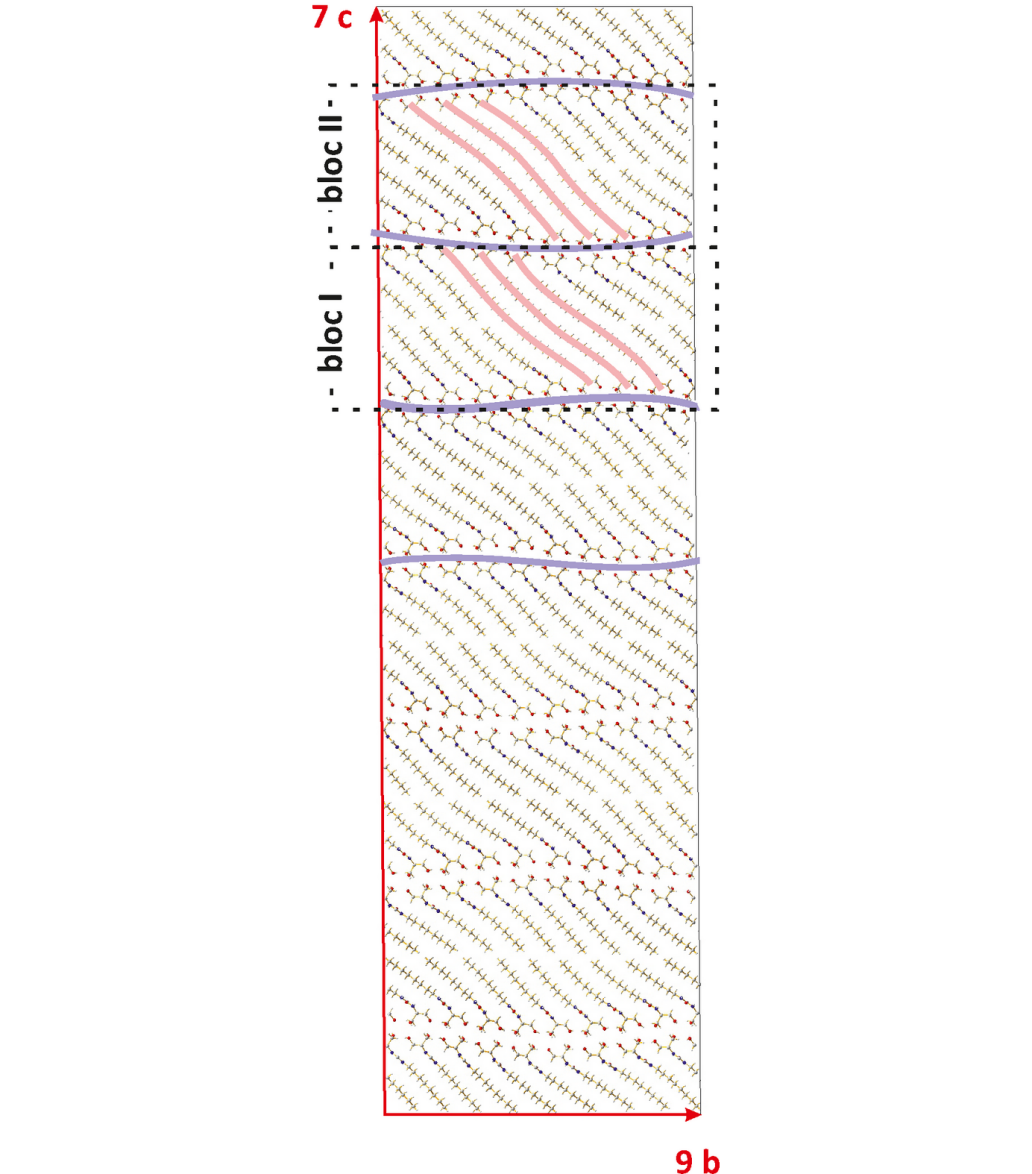
Projection along *a* of the (*a*,9*b*,7*c*) approximant unit cell of the structure of **2**. The structure can be regarded as an assembly of stacked blocks (*e.g.* bloc I and II), each two molecules thick. The C-(COH)_2_ groups form ribbons running along the *b* axis (highlighted by purple lines), with their undulation being out of phase between adjacent blocks. In contrast, the long hydro­carbon chains (highlighted by purple lines) exhibit in-phase undulations both within a block and between successive blocks.

**Figure 19:**
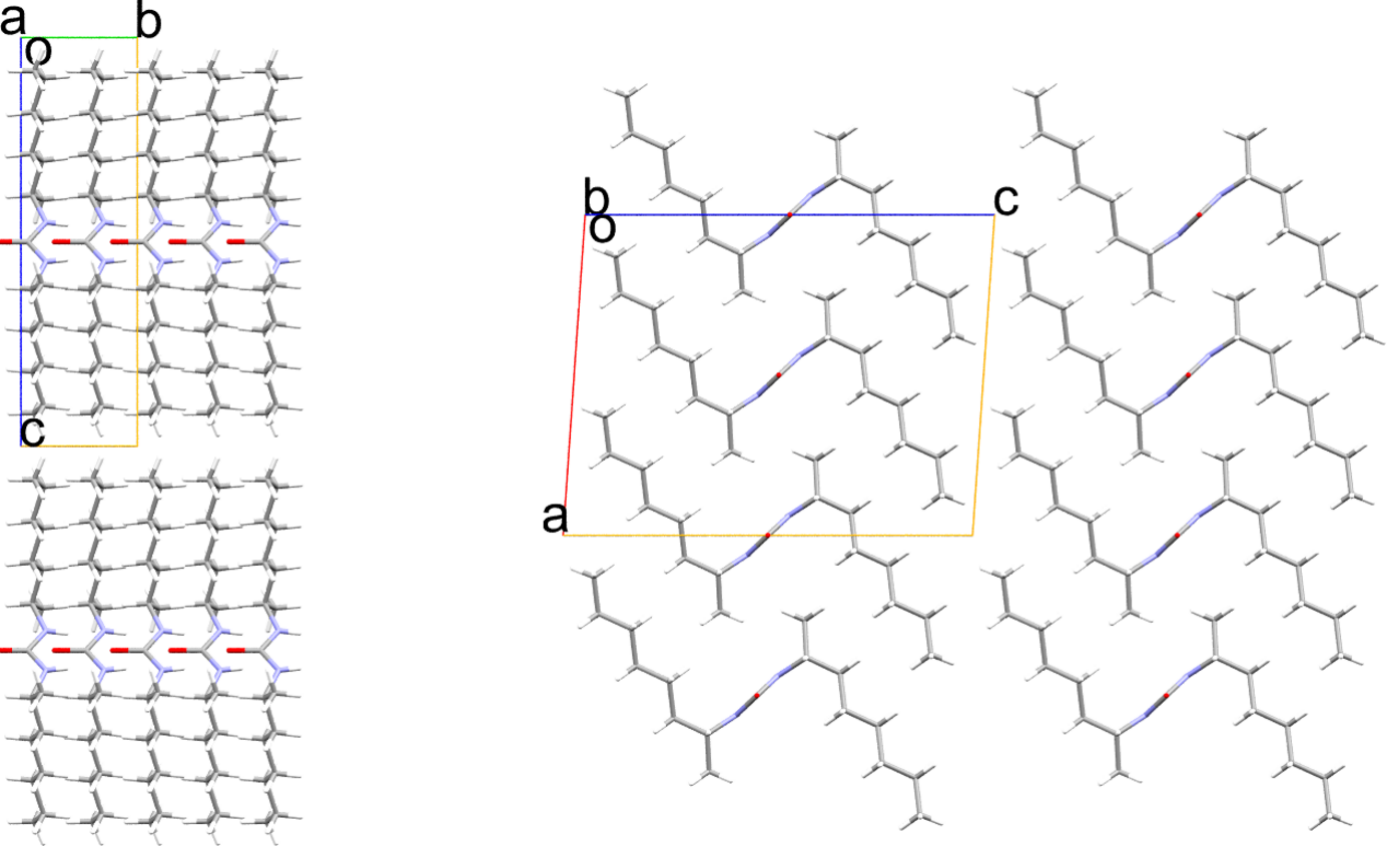
Projection along *a* (left) and *b* (right) of the structure of **3**.

**Figure 20:**
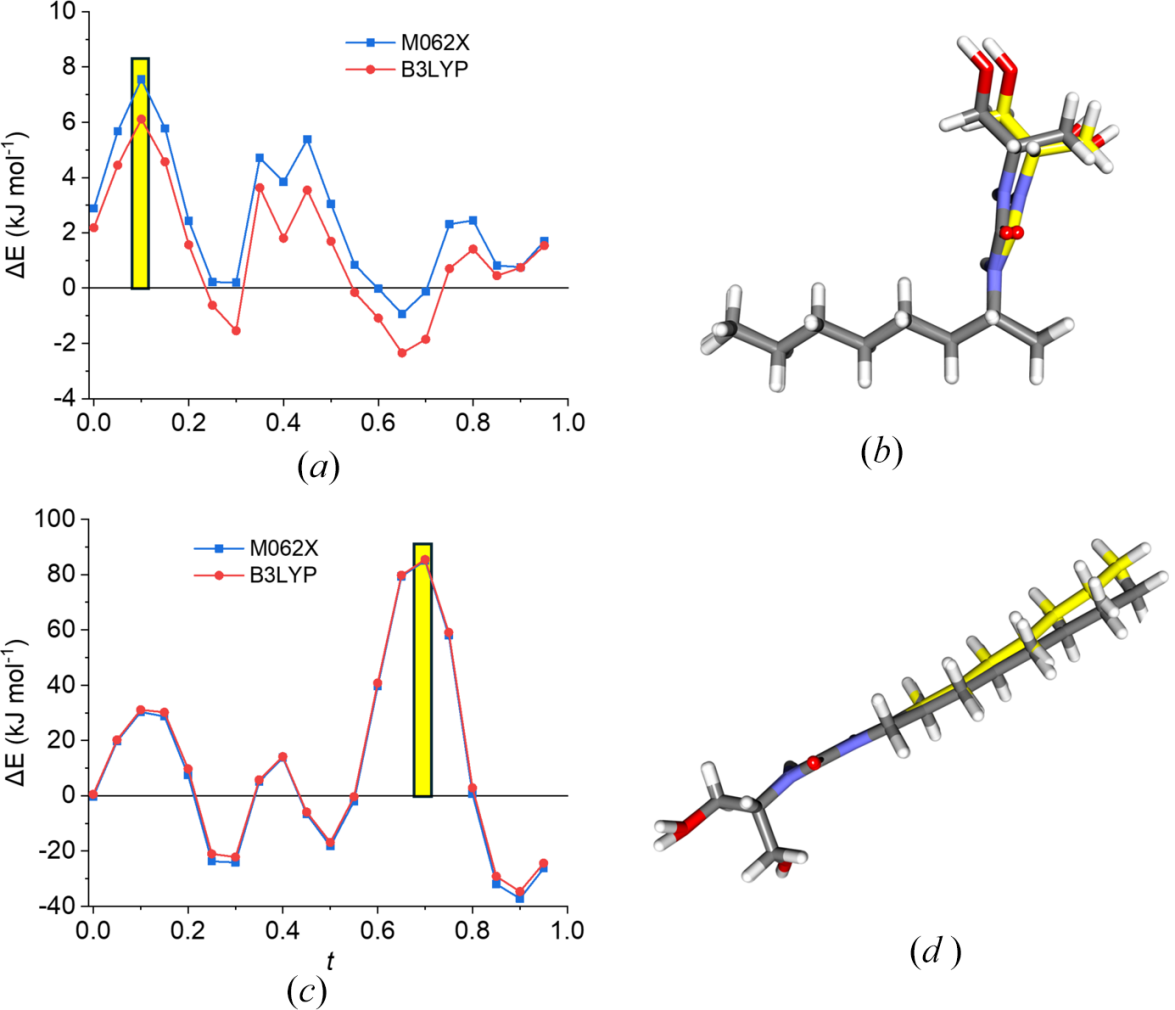
(*a*) Relative energy (kJ mol^−1^) with respect to the average structure as a function of the modulation parameter (*t*) for compound **1**. (*b*) Superimposed geometries of compound **1** corresponding to the energy maximum (yellow) and the average structure (gray). (*c*) Relative energy (kJ mol^−1^) with respect to the average structure as a function of *t* for compound **2**. (*d*) Superimposed geometries of compound **2** corresponding to the energy maximum (yellow) and the average structure (gray).

**Figure 21:**
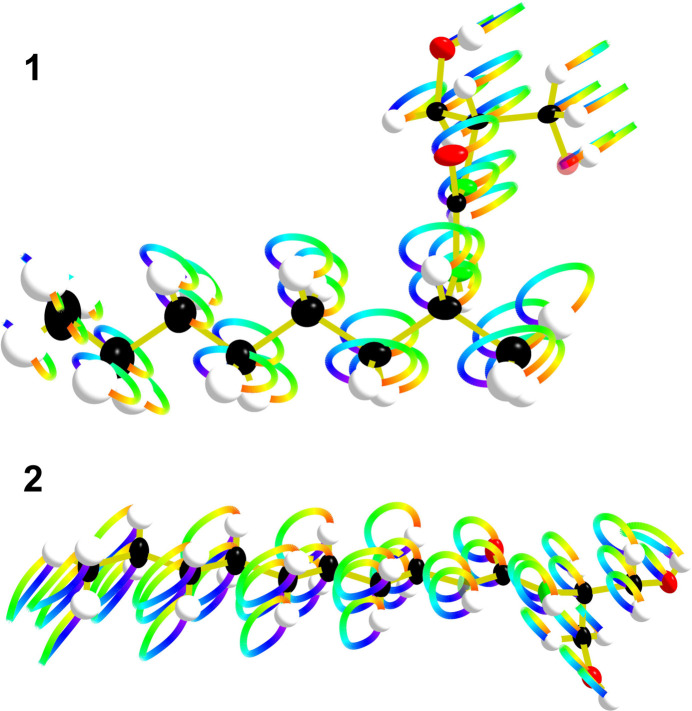
Lissajous visualizations of the effect of modulation on the individual atoms of **1** and **2**. Each colored ring traces the modulation path of an individual atom over one full period; the color progression indicates the internal phase *t*.

Python script to produce intensity statistics and Figs 5 and 6. DOI: 10.1107/S2052520626004932/dk5147sup3.exe

Input file for python script. DOI: 10.1107/S2052520626004932/dk5147sup4.txt

wB4rflxhWfA

CCDC references: 2553612, 2558679, 2558680

## Figures and Tables

**Figure 8:**
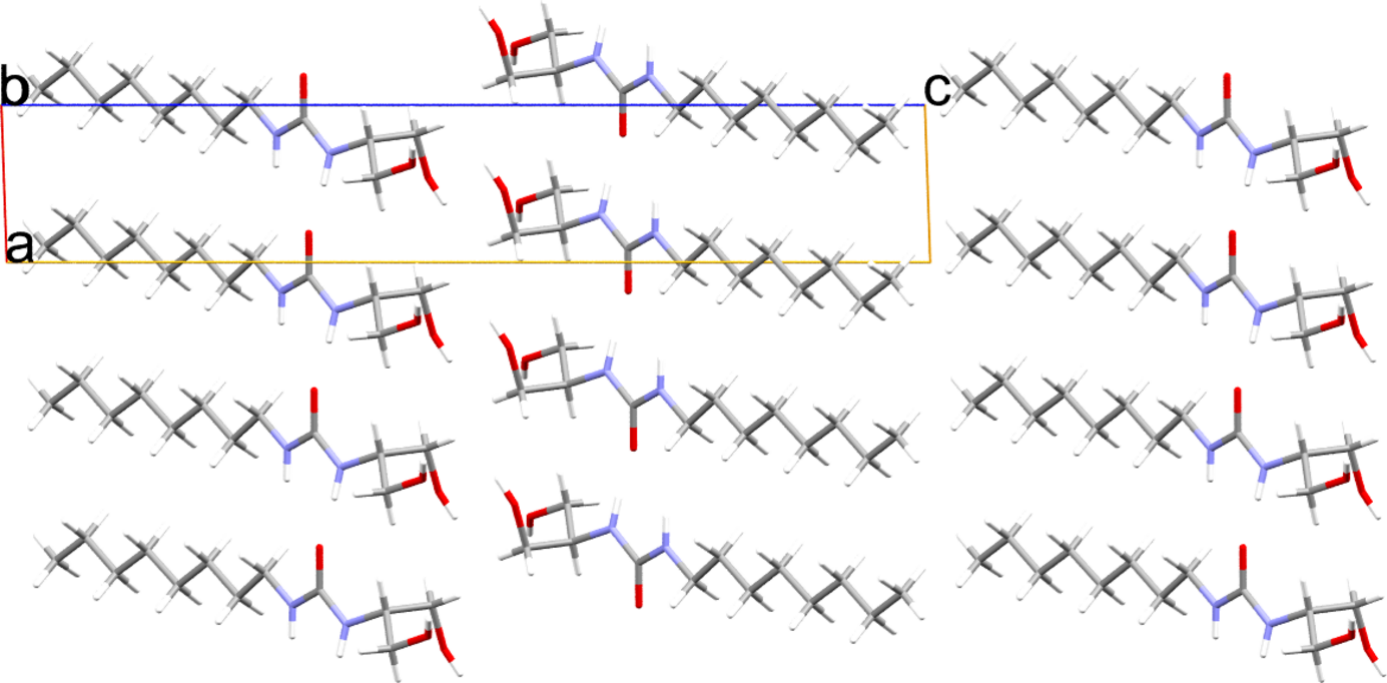
Packing of **2** along the ***b*** axis. The packing diagram has been drawn using the average structure.

**Table 1:** Experimental and refinement data for the three crystal structure determinations

	**1**	**2**	**3**
Crystal data			
Crystal system, space group	Monoclinic, *C*2(σ_1_0σ_3_)	Triclinic, *P*1(σ_1_ σ_2_σ_3_)	Monoclinic, *C*2
Wavevector	**q** = 0.2253 (2)a* + 0.5044 (4)c*	**q** = 0.00816 (14)a* + 0.1172 (2)b* + 0.5789 (5)c*	
*a*,*b*,*c* (Å)	11.516 (1), 4.619 (1), 26.775 (2)	4.6380 (4), 6.0367 (7), 25.779 (2)	12.667 (1), 4.5897 (4), 16.146 (2)
α,β, γ (°)	90, 91.410 (7), 90	89.802 (8), 88.106 (7), 71.190 (9)	90, 93.93 (1), 90
*V* (Å^3^)	1423.8 (4)	682.83 (12)	936.5 (2)
*Z*	4	2	2
			
Data collection			
Absorption correction	Multi-scan (*CrysAlisPro*)	Multi-scan (*CrysAlisPro*)	Multi-scan (*CrysAlisPro*)
*T*_min_, *T*_max_	0.82, 1.00	0.45, 1.00	0.45, 1.00
*N*[*I*>3σ(*I*)]	10348	10854	1146
*R*_int_	0.035	0.0912	0.034
			
Refinement			
*R*[*F*^2^ > 3σ(*F*^2^)], *wR*[*F*^2^ > 3σ(*F*^2^)], *S*	0.053, 0.074, 1.185	0.077, 0.110, 2.037	0.047, 0.112, 1.190
*R*[*F*^2^ > 3σ(*F*^2^)] per order (0, 1, 2, …)	0.047, 0.048, 0.057, 0.076	0.073, 0.075, 0.075, 0.079, 0.093	
*wR*[*F*^2^ > 3σ(*F*^2^)] per reflection order (0, 1, 2, …)	0.072, 0.068, 0.075, 0.094	0.116, 0.114, 0.104, 0.104, 0.113	
No. of reflections	18579	33499	1594
No. of parameters	557	657	94
No. of restraints	0	0	0
H-atom treatment	Constrained	Constrained	Constrained
Δρ_max_, Δρ_min_ (e Å^−3^)	0.27, −0.23	0.56, −0.60	0.11, −0.12
			

**Table 2:** Intensity statistics per reflection order for **1** and **2**

Order	*N*_ref_ (all)	*N*_ref_ [*I* > 2σ(*I*)]	〈*I*/σ(*I*)〉 (all)	〈*I*/σ(*I*)〉 [*I* > 2σ(*I*)]	%*I* (all)	%*I* [*I* > 2σ(*I*)]
**1**						
0	2494	2020	15.73	19.16	46	46
1	5018	4062	12.69	15.41	35	35
2	4986	3798	7.48	9.48	14	14
3	4570	2602	3.82	5.94	5	4
All	17068	12482	9.24	12.24	100	100
**2**						
0	3112	1728	6.34	10.74	32	33
1	6145	3524	6.44	10.62	38	39
2	6097	3242	5.68	9.95	18	18
3	5905	2733	4.37	8.52	8	8
4	5418	1968	2.95	6.75	4	3
All	26677	13195	5.09	9.46	100	100

## Data Availability

Raw data (data frames) are available upon request to the authors.

## References

[bb1] Agre, P. (2004). *Angew. Chem. Int. Ed.* **43**, 4278–4290. 10.1002/anie.200460804 15368374

[bb2] Anderson, K. M., Goeta, A. E. & Steed, J. W. (2008). *Cryst. Growth Des.* **8**, 2517–2524.

[bb3] Bakus, R. C. II, Atwood, D. A., Parkin, S., Brock, C. P. & Petricek, V. (2013). *Acta Cryst.* B**69**, 496–508. 10.1107/S205251921301782X 24056359

[bb4] Baudour, J. L. & Sanquer, M. (1983). *Acta Cryst.* B**39**, 75–84.

[bb5] Becke, A. D. (1993). *J. Chem. Phys.* **98**, 5648–5652.

[bb51] Bourhis, L. J., Dolomanov, O. V., Gildea, R. J., Howard, J. A. K. & Puschmann, H. (2015). *Acta Cryst.* A**71**, 59–75. 10.1107/S2053273314022207 PMC4283469 25537389

[bb6] Brock, C. P. (2016). *Acta Cryst.* B**72**, 807–821. 10.1107/S2052520616017297 27910831

[bb7] Bruno, I. J., Cole, J. C., Kessler, M., Luo, J., Motherwell, W. D. S., Purkis, L. H., Smith, B. R., Taylor, R., Cooper, R. I., Harris, S. E. & Orpen, A. G. (2004). *J. Chem. Inf. Comput. Sci.* **44**, 2133–2144. 10.1021/ci049780b 15554684

[bb8] Burla, M. C., Caliandro, R., Carrozzini, B., Cascarano, G. L., Cuocci, C., Giacovazzo, C., Mallamo, M., Mazzone, A. & Polidori, G. (2015). *J. Appl. Cryst.* **48**, 306–309.

[bb9] Cailleau, H., Baudour, J. L. & Zeyen, C. M. E. (1979). *Acta Cryst.* B**35**, 426–432.

[bb10] Cailleau, H., Moussa, F. & Mons, J. (1979). *Solid State Commun.* **31**, 521–524.

[bb11] Canadillas-Delgado, L., Mazzuca, L., Fabelo, O., Rodriguez-Velamazan, J. A. & Rodriguez-Carvajal, J. (2019). *IUCrJ* **6**, 105–115. 10.1107/S2052252518015026 PMC6327183 30713708

[bb12] Dzyabchenko, A. & Scheraga, H. A. (2004). *Acta Cryst.* B**60**, 228–237. 10.1107/S010876810400312X 15017097

[bb13] Elcoro, L., Pérez, O., Perez-Mato, J. M. & Petříček, V. (2012). *Acta Cryst.* B**68**, 341–355. 10.1107/S0108768112018095 22810904

[bb14] Forst, R., Jagodzinski, H., Boysen, H. & Frey, F. (1987). *Acta Cryst.* B**43**, 187–197.

[bb15] Frisch, M. J., Trucks, G. W., Schlegel, H. B., Scuseria, G. E., Robb, M. A., Cheeseman, J. R., Scalmani, G., Barone, V., Petersson, G. A., Nakatsuji, H., Li, X., Caricato, M., Marenich, A. V., Bloino, J., Janesko, B. G., Gomperts, R., Mennucci, B., Hratchian, H. P., Ortiz, J. V., Izmaylov, A. F., Sonnenberg, J. L., Williams-Young, D., Ding, F., Lipparini, F., Egidi, F., Goings, J., Peng, B., Petrone, A., Henderson, T., Ranasinghe, D., Zakrzewski, V. G., Gao, J., Rega, N., Zheng, G., Liang, W., Hada, M., Ehara, M., Toyota, K., Fukuda, R., Hasegawa, J., Ishida, M., Nakajima, T., Honda, Y., Kitao, O., Nakai, H., Vreven, T., Throssell, K., Montgomery, J. J. A., Peralta, J. E., Ogliaro, F., Bearpark, M. J., Heyd, J. J., Brothers, E. N., Kudin, K. N., Staroverov, V. N., Keith, T. A., Kobayashi, R., Normand, J., Raghavachari, K., Rendell, A. P., Burant, J. C., Iyengar, S. S., Tomasi, J., Cossi, M., Millam, J. M., Klene, M., Adamo, C., Cammi, R., Ochterski, J. W., Martin, R. L., Morokuma, K., Farkas, O., Foresman, J. B. & Fox, D. J. (2016). *Gaussian16*, Revision C.01. Gaussian Inc., Wallingford, CT, USA.

[bb16] Gabirondo-López, J., Gabirondo-López, I., Tasci, E. S. & Madariaga, G. (2024). *J. Appl. Cryst.* **57**, 1640–1649. 10.1107/S1600576724007908 PMC11460402 39387067

[bb17] Gaydamaka, A. A. & Rashchenko, S. V. (2024). *Acta Cryst.* B**80**, 676–681. 10.1107/S2052520624008710 39405198

[bb18] Groom, C. R., Bruno, I. J., Lightfoot, M. P. & Ward, S. C. (2016). *Acta Cryst.* B**72**, 171–179. 10.1107/S2052520616003954 PMC4822653 27048719

[bb19] Hanson, R. M. (2010). *J. Appl. Cryst.* **43**, 1250–1260.

[bb20] Huang, L.-B., Hardiagon, A., Kocsis, I., Jegu, C.-A., Deleanu, M., Gilles, A., van der Lee, A., Sterpone, F., Baaden, M. & Barboiu, M. (2021). *J. Am. Chem. Soc.* **143**, 4224–4233. 10.1021/jacs.0c11952 33635056

[bb21] Hübschle, C. B. & Dittrich, B. (2011). *J. Appl. Cryst.* **44**, 238–240. 10.1107/S0021889810042482 PMC3253731 22477783

[bb22] Johnstone, R. D. L., Ieva, M., Lennie, A. R., McNab, H., Pidcock, E., Warren, J. E. & Parsons, S. (2010). *CrystEngComm* **12**, 2520–2523.

[bb23] Kabsch, W. (2010*a*). *Acta Cryst.* D**66**, 125–132. 10.1107/S0907444909047337 PMC2815665 20124692

[bb24] Kabsch, W. (2010*b*). *Acta Cryst.* D**66**, 133–144. 10.1107/S0907444909047374 PMC2815666 20124693

[bb25] Lausi, A., Polentarutti, M., Onesti, S., Plaisier, J. R., Busetto, E., Bais, G., Barba, L., Cassetta, A., Campi, G., Lamba, D., Pifferi, A., Mande, S. C., Sarma, D. D., Sharma, S. M. & Paolucci, G. (2015). *Eur. Phys. J. Plus* **130**, 43.

[bb26] Lee, C., Yang, W. & Parr, R. G. (1988). *Phys. Rev. B* **37**, 785–789. 10.1103/physrevb.37.785 9944570

[bb27] Li, J., Pan, F., Geng, S., Lin, C., Palatinus, L., Allix, M., Kuang, X., Lin, J. & Sun, J. (2020). *Nat. Commun.* **11**, 4751. 10.1038/s41467-020-18481-x PMC7506534 32958759

[bb28] Lin, K., Zhou, Z., Liu, L., Ma, H., Chen, J., Deng, J., Sun, J., You, L., Kasai, H., Kato, K., Takata, M. & Xing, X. (2015). *J. Am. Chem. Soc.* **137**, 13468–13471. 10.1021/jacs.5b08230 26474121

[bb71] Lissajous, J. A. (1857). *Ann. Chim. Phys.* 3e série, **51**, 147–231.

[bb29] Mariette, C., Rabiller, P., Guérin, L., Ecolivet, C., Frantsuzov, I., Wang, B., Nichols, S. M., Bourges, P., Bosak, A., Chen, Y.-S., Hollingsworth, M. D. & Toudic, B. (2022). *Phys. Rev. B* **106**, 134109.

[bb70] Nguyen, T. L., Fowler, F. W. & Lauher, J. W. (2001). *J. Am. Chem. Soc.* **123**, 11057–11064. 10.1021/ja016635v 11686712

[bb30] Palatinus, L. (2004). *Acta Cryst.* A**60**, 604–610. 10.1107/S0108767304022433 15507744

[bb31] Palatinus, L. & Chapuis, G. (2007). *J. Appl. Cryst.* **40**, 786–790.

[bb32] Pavlyuk, V., Chumak, I., Akselrud, L., Lidin, S. & Ehrenberg, H. (2014). *Acta Cryst.* B**70**, 212–217. 10.1107/S2052520613030709 24675590

[bb33] Petříček, V., Dušek, M. & Palatinus, L. (2014). *Z. Kristallogr. Cryst. Mater.* **229**, 345–352.

[bb34] Petříček, V., Palatinus, L., Plášil, J. & Dušek, M. (2023). *Z. Kristallogr. Cryst. Mater.* **238**, 271–282.

[bb35] Rekis, T., Schönleber, A., Noohinejad, L., Tolkiehn, M., Paulmann, C. & van Smaalen, S. (2021). *Cryst. Growth Des.* **21**, 2324–2331.

[bb36] Rekis, T., Schönleber, A. & van Smaalen, S. (2020). *Acta Cryst.* B**76**, 18–27. 10.1107/S2052520619014938 PMC8788847 32831236

[bb50] Rigaku Oxford Diffraction (2012). *CrysAlis PRO*. Rigaku Oxford Diffraction,Yarnton, England.

[bb37] Schoenleber, A. (2011). *Z. Kristallogr.* **226**, 499–517.

[bb38] Schönleber, A. & Chapuis, G. (2004). *Acta Cryst.* B**60**, 108–120. 10.1107/S0108768103027605 14734850

[bb74] Schneider, T. R. & Sheldrick, G. M. (2002). *Acta Cryst.* D**58**, 1772–1779. 10.1107/s0907444902011678 12351820

[bb52] Sheldrick, G. M. (2008). *Acta Cryst.* A**64**, 112–122. 10.1107/S0108767307043930 18156677

[bb39] Steed, K. M. & Steed, J. W. (2015). *Chem. Rev.* **115**, 2895–2933. 10.1021/cr500564z 25675105

[bb40] Van Smaalen, S. & Harris, K. D. M. (1997). *Proc. R. Soc. London. Ser. A: Math. Phys. Eng. Sci.* **452**, 677–700.

[bb41] Weigend, F. & Ahlrichs, R. (2005). *Phys. Chem. Chem. Phys.* **7**, 3297–3305. 10.1039/b508541a 16240044

[bb42] Yamamoto, A. (1980). *Phys. Rev. B* **22**, 373–379.

[bb43] Zhao, Y. & Truhlar, D. G. (2008). *Theor. Chem. Acc.* **120**, 215–241.

[bb44] Zuñiga, F. J., Madariaga, G., Paciorek, W. A., Pérez-Mato, J. M., Ezpeleta, J. M. & Etxebarria, I. (1989). *Acta Cryst.* B**45**, 566–576.

