# Supplementary material for: Deciphering unusually large modulations in two related organic hy­droxy channel structures
Source: Acta Crystallogr B Struct Sci Cryst Eng Mater. 2026 Jun 10;82(Pt 4):375–90. doi: 10.1107/S2052520626004932 (PMC13470797; doi:10.1107/S2052520626004932)
Supplement: Supplementary file 1 [file b-82-00375-sup2.pdf]

Intensity statistics of 117 modulated structures.
Supplementary material for paper: 'Deciphering extreme modulations in two related organic hydroxy channel structures'

Arie van der Lee, Ioan Stroiu, Li-Bu Huang, Erol Licsandru, Istvan Kocsis, Mihail Deleanu, Christian Hübschle Mihail Barboiu and Olivier Pérez

---------------- November 2025 ----------------

i:inorganic structure
o:organic structure
mo:metal-organic structure
h:hybrid structure

Structure 1: https://doi.org/S2052520615024865 i

order	nref-all	nref-gt	i/s-all	i/s-gt	%int-all	%int-gt	falloff-fract
0	479	463	46.17	47.72	0.87	0.88	1.00
1	1523	1274	10.87	12.80	0.11	0.11	0.13
2	1301	750	3.54	5.44	0.01	0.01	0.02
all	3303	2487	13.10	17.08	1.00	1.00	1.00

Structure 2: https://doi.org/S2052520624008710 o

order	nref-all	nref-gt	i/s-all	i/s-gt	%int-all	%int-gt	falloff-fract
0	874	714	25.71	31.26	0.62	0.63	1.00
1	1718	1256	13.99	18.76	0.27	0.27	0.43
2	1590	1064	6.28	8.89	0.11	0.10	0.17
all	4182	3034	13.51	18.24	1.00	1.00	1.00

Structure 3: https://doi.org/S0108768106025791 h

order	nref-all	nref-gt	i/s-all	i/s-gt	%int-all	%int-gt	falloff-fract
0	7817	6555	13.46	15.92	0.79	0.79	1.00
1	15457	10505	7.71	11.03	0.19	0.19	0.24
2	15119	4150	1.96	5.75	0.02	0.02	0.03
all	38393	21210	6.62	11.51	1.00	1.00	1.00

Structure 4: https://doi.org/S2052520620003807 i

order	nref-all	nref-gt	i/s-all	i/s-gt	%int-all	%int-gt	falloff-fract
0	2122	2116	72.92	73.12	0.99	0.99	1.00
1	4853	3677	5.52	6.94	0.01	0.01	0.01
all	6975	5793	26.03	31.11	1.00	1.00	1.00

Structure 5: https://doi.org/S2052520620012640 i

order	nref-all	nref-gt	i/s-all	i/s-gt	%int-all	%int-gt	falloff-fract
0	186	151	24.52	29.93	0.84	0.86	1.00
1	261	162	7.49	11.37	0.15	0.14	0.17
2	288	46	1.49	3.61	0.01	0.00	0.02
all	735	359	9.45	18.19	1.00	1.00	1.00

Structure 6: https://doi.org/S205252061901494X i

order	nref-all	nref-gt	i/s-all	i/s-gt	%int-all	%int-gt	falloff-fract
0	107	106	78.94	79.67	0.94	0.94	1.00
1	174	164	56.80	60.18	0.06	0.06	0.07
2	202	155	10.95	13.88	0.00	0.00	0.00
3	161	122	18.21	23.73	0.00	0.00	0.00
all	644	547	36.45	42.71	1.00	1.00	1.00

Structure 7: https://doi.org/S2052520619016391 (a) i

order	nref-all	nref-gt	i/s-all	i/s-gt	%int-all	%int-gt	falloff-fract
0	259	243	65.61	69.85	0.66	0.66	1.00
1	431	350	37.71	46.22	0.30	0.30	0.45
2	367	207	9.92	16.90	0.04	0.04	0.06
3	302	66	1.72	4.84	0.00	0.00	0.01
all	1359	866	27.52	42.69	1.00	1.00	1.00

Structure 8: https://doi.org/S2052520619016391 (b) i

order	nref-all	nref-gt	i/s-all	i/s-gt	%int-all	%int-gt	falloff-fract
0	271	251	57.20	61.67	0.64	0.64	1.00
1	439	343	28.07	35.65	0.31	0.31	0.49
2	382	202	7.40	13.24	0.05	0.04	0.07
3	173	60	2.13	4.50	0.00	0.00	0.01
all	1265	856	24.52	35.81	1.00	1.00	1.00

Structure 9: https://doi.org/S2052520619016391 (c) i

order	nref-all	nref-gt	i/s-all	i/s-gt	%int-all	%int-gt	falloff-fract
0	256	232	62.23	68.56	0.61	0.62	1.00
1	417	334	33.63	41.74	0.32	0.32	0.53
2	363	200	10.19	17.80	0.06	0.05	0.09
3	170	70	3.02	6.12	0.01	0.00	0.01
all	1206	836	28.33	40.47	1.00	1.00	1.00

Structure 10: https://doi.org/S2052520619013155 (a) mo

order	nref-all	nref-gt	i/s-all	i/s-gt	%int-all	%int-gt	falloff-fract
0	1747	1633	33.27	35.52	0.97	0.97	1.00
1	2990	1748	4.15	6.43	0.03	0.03	0.04
all	4737	3381	14.89	20.48	1.00	1.00	1.00

Structure 11: https://doi.org/S2052520619013155 (b) mo

order	nref-all	nref-gt	i/s-all	i/s-gt	%int-all	%int-gt	falloff-fract
0	1746	1686	43.33	44.83	0.95	0.95	1.00
1	3095	2335	10.87	14.08	0.05	0.05	0.05
all	4841	4021	22.58	26.98	1.00	1.00	1.00

Structure 12: https://doi.org/S2052520617009398 o

order	nref-all	nref-gt	i/s-all	i/s-gt	%int-all	%int-gt	falloff-fract
0	1895	1232	29.44	44.79	0.86	0.87	1.00
1	3348	2070	19.25	30.56	0.14	0.13	0.16
all	5243	3302	22.94	35.87	1.00	1.00	1.00

Structure 13: https://doi.org/S2052520615004084 o

order	nref-all	nref-gt	i/s-all	i/s-gt	%int-all	%int-gt	falloff-fract
0	5900	5717	23.97	24.70	1.00	1.00	1.00
1	7930	3484	3.03	5.70	0.00	0.00	0.00
all	13830	9201	11.96	17.51	1.00	1.00	1.00

Structure 14: https://doi.org/S205252061601578X i

order	nref-all	nref-gt	i/s-all	i/s-gt	%int-all	%int-gt	falloff-fract
0	1839	1832	186.35	187.06	0.86	0.86	1.00
1	3677	3417	49.04	52.70	0.13	0.13	0.15
2	3416	2396	7.25	9.94	0.01	0.01	0.01
all	8932	7645	61.33	71.49	1.00	1.00	1.00

Structure 15: https://doi.org/S2052520619013623 i

order	nref-all	nref-gt	i/s-all	i/s-gt	%int-all	%int-gt	falloff-fract
0	1467	1377	85.29	90.82	0.85	0.85	1.00
1	1838	1470	29.58	36.81	0.12	0.12	0.14
2	1813	1051	7.91	13.18	0.03	0.03	0.03
all	5118	3898	37.87	49.52	1.00	1.00	1.00

Structure 16: https://doi.org/S2052252517000264 i

order	nref-all	nref-gt	i/s-all	i/s-gt	%int-all	%int-gt	falloff-fract
0	409	377	8.39	9.04	0.92	0.92	1.00
1	662	509	6.14	7.67	0.06	0.06	0.07
2	162	125	5.33	6.64	0.01	0.01	0.01
3	76	62	8.77	10.50	0.00	0.00	0.00
all	1309	1073	6.90	8.19	1.00	1.00	1.00

Structure 17: https://doi.org/S2052520618003669 mo

```
 order nref-all nref-gt i/s-all i/s-gt    %int-all %int-gt   falloff-fract
     0   1791   1791    31.42    31.42     0.84     0.84     1.00
     1   2698   2698    14.35    14.35     0.16     0.16     0.20
   all   4489   4489    21.16    21.16     1.00     1.00     1.00
Structure 18: https://doi.org/S2052520617002657 i
 order nref-all nref-gt i/s-all i/s-gt    %int-all %int-gt   falloff-fract
     0   1463   1424    53.59    55.02     0.92     0.92     1.00
     1   2441   2029    12.01    14.23     0.06     0.06     0.07
     2   2384   1756     5.80     7.47     0.02     0.02     0.02
   all   6288   5209    19.33    23.10     1.00     1.00     1.00
Structure 19: https://doi.org/S2052520617007156 i
 order nref-all nref-gt i/s-all i/s-gt    %int-all %int-gt   falloff-fract
     0   2306   1948    28.48    33.52     0.89     0.90     1.00
     1   4475   3185     6.75     9.06     0.09     0.09     0.10
     2   4268   1979     2.58     4.34     0.02     0.01     0.02
   all  11049   7112     9.68    14.44     1.00     1.00     1.00
Structure 20: https://doi.org/S2052520617001652 i
 order nref-all nref-gt i/s-all i/s-gt    %int-all %int-gt   falloff-fract
     0    531    478    35.80    39.68     0.93     0.94     1.00
     1    234     97     2.54     4.83     0.07     0.06     0.08
   all    765    575    25.63    33.80     1.00     1.00     1.00
Structure 21: https://doi.org/S2052520619007340 i
 order nref-all nref-gt i/s-all i/s-gt    %int-all %int-gt   falloff-fract
     0    479    409    32.31    37.72     0.78     0.78     1.00
     1    748    612    12.67    15.32     0.22     0.22     0.28
   all   1227   1021    20.34    24.29     1.00     1.00     1.00
Structure 22: https://doi.org/S2052252518015026 mo
 order nref-all nref-gt i/s-all i/s-gt    %int-all %int-gt   falloff-fract
     0    734    732    83.53    83.76     0.90     0.90     1.00
     1   1256   1067    22.25    26.00     0.10     0.10     0.11
     2    906    263     2.16     5.13     0.00     0.00     0.00
   all   2896   2062    31.50    43.84     1.00     1.00     1.00
Structure 23: https://doi.org/S2052520620015929 o
 order nref-all nref-gt i/s-all i/s-gt    %int-all %int-gt   falloff-fract
     0   2533   2531    67.07    67.12     0.89     0.89     1.00
     1   5095   5018    41.98    42.60     0.11     0.11     0.12
     2   4974   3315     5.93     8.39     0.00     0.00     0.00
     3   4650   1827     2.46     4.88     0.00     0.00     0.00
   all  17252  12691    24.62    33.12     1.00     1.00     1.00
Structure 24: https://doi.org/S205252062100370X i
 order nref-all nref-gt i/s-all i/s-gt    %int-all %int-gt   falloff-fract
     0     52     52    34.97    34.97     0.66     0.66     1.00
     1    105    104    28.08    28.33     0.29     0.29     0.44
     2    105     95    24.52    26.97     0.05     0.05     0.07
   all    262    251    28.02    29.19     1.00     1.00     1.00
Structure 25: https://doi.org/S2052520615019733 (a) i
 order nref-all nref-gt i/s-all i/s-gt    %int-all %int-gt   falloff-fract
     0    885    880    28.42    28.57     0.81     0.81     1.00
     1   1773   1714     9.93    10.22     0.18     0.18     0.22
     2   1554    867     2.53     3.59     0.01     0.01     0.02
   all   4212   3461    11.08    13.23     1.00     1.00     1.00
Structure 26: https://doi.org/S2052520615019733 (b) i
 order nref-all nref-gt i/s-all i/s-gt    %int-all %int-gt   falloff-fract
     0   1990   1988    28.81    28.84     0.94     0.94     1.00
```

1	1972	1888	9.39	9.76	0.05	0.05	0.06
2	1965	969	2.32	3.71	0.00	0.00	0.00
all	5927	4845	13.57	16.38	1.00	1.00	1.00

Structure 27: https://doi.org/S010876810201741X i

order	nref-all	nref-gt	i/s-all	i/s-gt	%int-all	%int-gt	falloff-fract
0	3712	3626	20.11	20.57	0.99	0.99	1.00
1	1382	1262	11.23	12.21	0.01	0.01	0.01
all	5094	4888	17.70	18.41	1.00	1.00	1.00

Structure 28: https://doi.org/S010876810201772X o

order	nref-all	nref-gt	i/s-all	i/s-gt	%int-all	%int-gt	falloff-fract
0	1850	1837	23.43	23.58	0.70	0.70	1.00
1	2396	2367	15.23	15.40	0.30	0.30	0.43
all	4246	4204	18.80	18.97	1.00	1.00	1.00

Structure 29: https://doi.org/S2052252524011060 i

order	nref-all	nref-gt	i/s-all	i/s-gt	%int-all	%int-gt	falloff-fract
0	839	774	40.52	43.85	0.76	0.77	1.00
1	1575	1058	12.99	18.89	0.21	0.21	0.27
2	1541	713	3.83	7.26	0.03	0.03	0.04
all	3955	2545	15.26	23.22	1.00	1.00	1.00

Structure 30: https://doi.org/10.1021-jacs.4c13545 i

order	nref-all	nref-gt	i/s-all	i/s-gt	%int-all	%int-gt	falloff-fract
0	321	312	37.42	38.47	0.31	0.31	1.00
1	631	610	35.47	36.65	0.37	0.37	1.22
2	618	591	25.37	26.48	0.20	0.20	0.64
3	607	540	14.92	16.64	0.09	0.09	0.30
4	561	401	7.37	9.90	0.03	0.03	0.10
all	2738	2454	23.11	25.66	1.00	1.00	1.00

Structure 31: https://doi.org/S0108768102021195 i

order	nref-all	nref-gt	i/s-all	i/s-gt	%int-all	%int-gt	falloff-fract
0	3328	3298	33.02	33.31	0.66	0.66	1.00
1	5420	5360	14.92	15.07	0.28	0.28	0.43
2	3079	2932	8.61	8.99	0.06	0.06	0.09
all	11827	11590	18.37	18.72	1.00	1.00	1.00

Structure 32: https://doi.org/S0108768103009017 h

order	nref-all	nref-gt	i/s-all	i/s-gt	%int-all	%int-gt	falloff-fract
0	814	800	28.74	29.22	0.71	0.71	1.00
1	1244	1215	25.20	25.78	0.24	0.24	0.34
2	1032	997	18.40	19.01	0.05	0.05	0.07
3	594	566	11.71	12.24	0.01	0.01	0.01
4	329	303	7.95	8.52	0.00	0.00	0.00
5	111	87	4.33	5.27	0.00	0.00	0.00
all	4124	3968	20.32	21.07	1.00	1.00	1.00

Structure 33: https://doi.org/S0108768103019694 i

order	nref-all	nref-gt	i/s-all	i/s-gt	%int-all	%int-gt	falloff-fract
0	195	174	143.44	160.65	0.56	0.56	1.00
1	363	328	71.02	78.53	0.32	0.32	0.57
2	369	285	23.84	30.66	0.08	0.08	0.14
3	351	241	11.00	15.63	0.03	0.03	0.05
4	337	115	2.31	5.52	0.01	0.00	0.01
5	333	108	2.36	6.08	0.01	0.00	0.01
all	1948	1251	34.89	53.96	1.00	1.00	1.00

Structure 34: https://doi.org/S0108768104000874 o

order	nref-all	nref-gt	i/s-all	i/s-gt	%int-all	%int-gt	falloff-fract
0	1451	1300	52.80	58.85	0.97	0.97	1.00

1	2442	1253	5.18	9.47	0.03	0.03	0.03
all	3893	2553	22.93	34.61	1.00	1.00	1.00

Structure 35: https://doi.org/S0108768105001655 i

order	nref-all	nref-gt	i/s-all	i/s-gt	%int-all	%int-gt	falloff-fract
0	133	132	22.18	22.33	0.24	0.24	1.00
1	267	265	19.34	19.48	0.37	0.37	1.52
2	209	208	16.94	17.02	0.22	0.22	0.90
3	178	178	13.37	13.37	0.12	0.12	0.48
4	138	136	10.90	11.04	0.06	0.06	0.25
all	925	919	16.80	16.90	1.00	1.00	1.00

Structure 36: https://doi.org/S0108768105026480 i

order	nref-all	nref-gt	i/s-all	i/s-gt	%int-all	%int-gt	falloff-fract
0	358	316	28.58	32.27	0.90	0.90	1.00
1	564	452	13.70	16.91	0.10	0.10	0.11
all	922	768	19.48	23.23	1.00	1.00	1.00

Structure 37: https://doi.org/S0108768105012632 i

order	nref-all	nref-gt	i/s-all	i/s-gt	%int-all	%int-gt	falloff-fract
0	832	828	22.49	22.59	0.57	0.57	1.00
1	1598	1596	20.03	20.06	0.38	0.38	0.67
2	666	651	8.82	8.99	0.03	0.03	0.05
3	399	378	9.83	10.32	0.02	0.02	0.03
all	3495	3453	17.32	17.51	1.00	1.00	1.00

Structure 38: https://doi.org/S010876810501726X i

order	nref-all	nref-gt	i/s-all	i/s-gt	%int-all	%int-gt	falloff-fract
0	753	750	28.55	28.66	0.75	0.76	1.00
1	1176	1143	17.37	17.82	0.15	0.15	0.19
2	574	525	11.32	12.28	0.03	0.03	0.04
3	908	858	12.77	13.45	0.07	0.07	0.10
all	3411	3276	17.59	18.27	1.00	1.00	1.00

Structure 39: https://doi.org/S2052520616014049 i

order	nref-all	nref-gt	i/s-all	i/s-gt	%int-all	%int-gt	falloff-fract
0	1389	1244	31.32	34.86	0.96	0.96	1.00
1	1599	830	4.09	7.23	0.02	0.02	0.02
2	1833	764	3.18	6.74	0.01	0.01	0.01
3	1638	734	2.76	5.36	0.01	0.01	0.01
4	968	496	2.83	4.82	0.00	0.00	0.00
all	7427	4068	8.50	14.96	1.00	1.00	1.00

Structure 40: https://doi.org/S0108768106010238 i

order	nref-all	nref-gt	i/s-all	i/s-gt	%int-all	%int-gt	falloff-fract
0	283	277	21.74	22.19	0.80	0.80	1.00
1	495	459	18.01	19.37	0.17	0.17	0.21
2	568	433	9.30	11.97	0.02	0.02	0.03
3	493	220	2.93	5.76	0.00	0.00	0.00
4	565	182	1.73	3.97	0.00	0.00	0.00
all	2404	1571	9.47	14.14	1.00	1.00	1.00

Structure 41: https://doi.org/S0108768105039212 i

order	nref-all	nref-gt	i/s-all	i/s-gt	%int-all	%int-gt	falloff-fract
0	254	254	14.35	14.35	0.45	0.45	1.00
1	213	211	11.26	11.36	0.17	0.17	0.37
2	355	351	11.28	11.40	0.22	0.22	0.48
3	154	152	10.33	10.45	0.09	0.09	0.19
4	150	149	9.62	9.68	0.08	0.08	0.17
all	1126	1117	11.62	11.70	1.00	1.00	1.00

Structure 42: J. Rouquette & O. Pérez (private communication/unpublished

results, 2025) i

order	nref-all	nref-gt	i/s-all	i/s-gt	%int-all	%int-gt	falloff-fract
0	2376	2375	39.06	39.07	0.95	0.95	1.00
1	4456	4142	8.38	8.92	0.05	0.05	0.05
all	6832	6517	19.05	19.91	1.00	1.00	1.00

Structure 43: https://doi.org/S0108768188007001 i

order	nref-all	nref-gt	i/s-all	i/s-gt	%int-all	%int-gt	falloff-fract
0	422	422	29.81	29.81	0.84	0.84	1.00
1	693	678	25.81	26.35	0.12	0.12	0.15
2	826	733	17.01	19.04	0.03	0.03	0.04
3	631	453	9.43	12.74	0.01	0.01	0.01
4	726	452	6.02	9.10	0.00	0.00	0.00
5	519	269	3.91	6.71	0.00	0.00	0.00
6	553	127	1.37	3.12	0.00	0.00	0.00
7	389	31	0.95	2.64	0.00	0.00	0.00
all	4759	3165	12.18	17.87	1.00	1.00	1.00

Structure 44: https://doi.org/S0108768110035792 i

order	nref-all	nref-gt	i/s-all	i/s-gt	%int-all	%int-gt	falloff-fract
0	2479	2417	39.53	40.52	0.91	0.91	1.00
1	4513	3671	10.76	12.99	0.09	0.09	0.10
all	6992	6088	20.96	23.92	1.00	1.00	1.00

Structure 45: https://doi.org/S0108768106030382 o

order	nref-all	nref-gt	i/s-all	i/s-gt	%int-all	%int-gt	falloff-fract
0	898	730	13.53	16.50	0.97	0.97	1.00
1	1426	469	3.55	9.73	0.03	0.03	0.03
all	2324	1199	7.41	13.85	1.00	1.00	1.00

Structure 46: https://doi.org/S2052520616005643 i

order	nref-all	nref-gt	i/s-all	i/s-gt	%int-all	%int-gt	falloff-fract
0	778	778	36.11	36.11	0.35	0.35	1.00
1	1608	1608	34.34	34.34	0.44	0.44	1.26
2	1207	1207	30.84	30.84	0.18	0.18	0.50
3	601	601	24.49	24.49	0.04	0.04	0.12
all	4194	4194	32.25	32.25	1.00	1.00	1.00

Structure 47: https://doi.org/S205252062300817X i

order	nref-all	nref-gt	i/s-all	i/s-gt	%int-all	%int-gt	falloff-fract
0	981	761	12.39	15.84	0.99	1.00	1.00
1	85	66	6.09	7.48	0.01	0.00	0.01
all	1066	827	11.89	15.18	1.00	1.00	1.00

Structure 48: https://doi.org/S2052520623000999 i

order	nref-all	nref-gt	i/s-all	i/s-gt	%int-all	%int-gt	falloff-fract
0	1614	1550	159.07	165.59	1.00	1.00	1.00
1	2983	1597	3.35	5.48	0.00	0.00	0.00
all	4597	3147	58.02	84.34	1.00	1.00	1.00

Structure 49: https://doi.org/S2053229624007009 o

order	nref-all	nref-gt	i/s-all	i/s-gt	%int-all	%int-gt	falloff-fract
0	2551	2515	57.74	58.55	0.44	0.44	1.00
1	5383	5053	20.00	21.24	0.06	0.06	0.13
2	5383	5053	20.00	21.24	0.06	0.06	0.13
3	2551	2515	57.74	58.55	0.44	0.44	1.00
all	15868	15136	32.14	33.64	1.00	1.00	1.00

Structure 50: https://doi.org/S205251921301782X o

order	nref-all	nref-gt	i/s-all	i/s-gt	%int-all	%int-gt	falloff-fract
0	1767	1554	28.70	32.50	0.40	0.40	1.00
1	3494	3004	30.00	34.74	0.37	0.37	0.94

```
     2   3428   2817    17.47    21.05     0.16     0.16     0.41
     3   3124   2235     8.17    11.04     0.05     0.05     0.14
     4   2868   1510     4.03     6.83     0.02     0.02     0.04
   all  14681  11120    17.20    22.40     1.00     1.00     1.00
Structure 51: https://doi.org/S0108768109020898 i
 order nref-all nref-gt i/s-all i/s-gt    %int-all %int-gt   falloff-fract
     0   1952   1952    15.67    15.67     0.84     0.84     1.00
     1   2145   2145     9.11     9.11     0.16     0.16     0.19
   all   4097   4097    12.23    12.23     1.00     1.00     1.00
Structure 52: https://doi.org/S0108768110050822 i
 order nref-all nref-gt i/s-all i/s-gt    %int-all %int-gt   falloff-fract
     0   4925   4771    65.82    67.91     0.97     0.97     1.00
     1  17174   9007     5.59     9.83     0.03     0.03     0.03
   all  22099  13778    19.01    29.94     1.00     1.00     1.00
Structure 53: https://doi.org/S0108768100011332 i
 order nref-all nref-gt i/s-all i/s-gt    %int-all %int-gt   falloff-fract
     0    263    246    18.59    19.81     0.87     0.90     1.00
     1   1963   1034     4.38     7.42     0.13     0.10     0.14
   all   2226   1280     6.05     9.80     1.00     1.00     1.00
Structure 54: https://doi.org/S0108768109053713 i
 order nref-all nref-gt i/s-all i/s-gt    %int-all %int-gt   falloff-fract
     0    357    172     7.39    14.54     0.35     0.35     1.00
     1    652    347     6.71    11.94     0.24     0.24     0.69
     2    654    339     6.77    12.29     0.19     0.19     0.55
     3    462    211     6.08    12.39     0.11     0.11     0.31
     4    351    185     5.89    10.43     0.08     0.08     0.23
     5    150     69     5.49    11.00     0.03     0.03     0.08
     6     46     26     6.03    10.11     0.01     0.01     0.02
   all   2672   1349     6.52    12.14     1.00     1.00     1.00
Structure 55: https://doi.org/S0108768109027177 i
 order nref-all nref-gt i/s-all i/s-gt    %int-all %int-gt   falloff-fract
     0    942    925    28.52    29.03     0.75     0.75     1.00
     1   3572   3368    12.78    13.48     0.20     0.20     0.26
     2   1845   1665     7.81     8.53     0.05     0.05     0.07
   all   6359   5958    13.67    14.51     1.00     1.00     1.00
Structure 56: https://doi.org/S2052519213014231 i
 order nref-all nref-gt i/s-all i/s-gt    %int-all %int-gt   falloff-fract
     0   2035   1855    95.34   104.47     0.98     0.99     1.00
     1   4013   2168     3.45     5.51     0.02     0.01     0.02
   all   6048   4023    34.36    51.14     1.00     1.00     1.00
Structure 57: https://doi.org/S205252061401806X i
 order nref-all nref-gt i/s-all i/s-gt    %int-all %int-gt   falloff-fract
     0     95     90    41.90    44.19     0.94     0.94     1.00
     1    149    131    10.83    12.15     0.05     0.05     0.05
     2    140      8     0.99     2.97     0.00     0.00     0.00
     3    145     83     2.98     4.42     0.01     0.00     0.01
   all    529    312    11.65    19.10     1.00     1.00     1.00
Structure 58: https://doi.org/S205252061401049X i
 order nref-all nref-gt i/s-all i/s-gt    %int-all %int-gt   falloff-fract
     0   1048   1013    30.59    31.61     0.95     0.95     1.00
     1   1731   1230    11.90    16.39     0.05     0.05     0.05
     2   1281    104     0.86     3.10     0.00     0.00     0.00
   all   4060   2347    13.24    22.37     1.00     1.00     1.00
Structure 59: https://doi.org/S2052519213003126 i
```

```
 order nref-all nref-gt i/s-all i/s-gt    %int-all %int-gt   falloff-fract
     0    517    492    37.72    39.58     0.20     0.20     1.00
     1   1107   1081    33.35    34.12     0.06     0.06     0.31
     2   1043   1020    39.22    40.08     0.19     0.19     0.94
     3   1122   1096    31.92    32.66     0.06     0.06     0.31
     4   1035    994    38.33    39.87     0.35     0.35     1.76
     5   1106   1077    30.96    31.77     0.06     0.06     0.33
     6    520    497    38.00    39.71     0.08     0.08     0.39
   all   6450   6257    35.16    36.22     1.00     1.00     1.00
Structure 60: https://doi.org/S0108768110040802 i
 order nref-all nref-gt i/s-all i/s-gt    %int-all %int-gt   falloff-fract
     0   1711   1550    28.65    31.52     0.83     0.84     1.00
     1   2890   2017    13.48    18.94     0.15     0.15     0.18
     2   2434    627     2.15     6.22     0.01     0.01     0.01
   all   7035   4194    13.25    21.69     1.00     1.00     1.00
Structure 61: https://doi.org/S0108768106039462 i
 order nref-all nref-gt i/s-all i/s-gt    %int-all %int-gt   falloff-fract
     0   1171   1171    71.60    71.60     0.81     0.81     1.00
     1   2079   1871    22.09    24.44     0.15     0.15     0.19
     2   1481   1179     9.66    11.88     0.03     0.03     0.04
     3    782    502     5.34     7.81     0.01     0.01     0.01
     4    126     91     7.36     9.84     0.00     0.00     0.00
     6      3      3     4.81     4.81     0.00     0.00     0.00
     7      4      1     2.78     6.92     0.00     0.00     0.00
   all   5646   4818    26.43    30.81     1.00     1.00     1.00
Structure 62: https://doi.org/S205252062400146X i
 order nref-all nref-gt i/s-all i/s-gt    %int-all %int-gt   falloff-fract
     0   2432   1969    13.14    15.97     0.76     0.77     1.00
     1   4443   2815     4.24     6.08     0.24     0.23     0.31
   all   6875   4784     7.39    10.15     1.00     1.00     1.00
Structure 63: https://doi.org/S2052520623000215 i
 order nref-all nref-gt i/s-all i/s-gt    %int-all %int-gt   falloff-fract
     0   4364   4270    18.05    18.42     0.97     0.97     1.00
     1   4031   2452     3.41     4.94     0.03     0.03     0.03
   all   8395   6722    11.02    13.50     1.00     1.00     1.00
Structure 64: https://doi.org/S2052252522003888 i
 order nref-all nref-gt i/s-all i/s-gt    %int-all %int-gt   falloff-fract
     0    348    348    56.68    56.68     0.93     0.93     1.00
     1    566    533     9.95    10.49     0.07     0.07     0.07
   all    914    881    27.74    28.73     1.00     1.00     1.00
Structure 65: https://doi.org/S205252061600874X i
 order nref-all nref-gt i/s-all i/s-gt    %int-all %int-gt   falloff-fract
     0    312    312   611.30   611.30     0.90     0.90     1.00
     1    474    418    47.08    53.22     0.10     0.10     0.12
   all    786    730   271.05   291.74     1.00     1.00     1.00
Structure 66: https://doi.org/S2052520619017128 i
 order nref-all nref-gt i/s-all i/s-gt    %int-all %int-gt   falloff-fract
     0   2425   2318    97.78   102.25     0.88     0.89     1.00
     1   4852   3881    22.04    27.34     0.11     0.11     0.12
     2   4542   2107     3.85     7.43     0.01     0.01     0.01
   all  11819   8306    30.59    43.19     1.00     1.00     1.00
Structure 67: https://doi.org/S2052519213029436 i
 order nref-all nref-gt i/s-all i/s-gt    %int-all %int-gt   falloff-fract
     0   1564   1538    10.45    10.61     0.91     0.91     1.00
```

```
     1   1476   1347     8.52     9.26     0.08     0.08     0.09
     2    738    517     5.62     7.74     0.00     0.00     0.00
   all   3778   3402     8.75     9.64     1.00     1.00     1.00
Structure 68: https://doi.org/S2052520616020680 i
 order nref-all nref-gt i/s-all i/s-gt    %int-all %int-gt   falloff-fract
     0  16434   9635     4.87     7.71     0.90     0.92     1.00
     1  31424   6083     1.17     3.97     0.10     0.08     0.11
   all  47858  15718     2.44     6.26     1.00     1.00     1.00
Structure 69: https://doi.org/S2052520615022076 i
 order nref-all nref-gt i/s-all i/s-gt    %int-all %int-gt   falloff-fract
     0   1564   1538    10.45    10.61     0.91     0.91     1.00
     1   1476   1347     8.52     9.26     0.08     0.08     0.09
     2    738    517     5.62     7.74     0.00     0.00     0.00
   all   3778   3402     8.75     9.64     1.00     1.00     1.00
Structure 70: https://doi.org/S0108768111013814 i
 order nref-all nref-gt i/s-all i/s-gt    %int-all %int-gt   falloff-fract
     0   1825   1825    34.71    34.71     0.90     0.90     1.00
     1   2617   2617    32.16    32.16     0.10     0.10     0.11
     2   1088   1088     8.13     8.13     0.00     0.00     0.00
     3    392    392     6.97     6.97     0.00     0.00     0.00
     4     86     86     4.19     4.19     0.00     0.00     0.00
     5    237    237     5.69     5.69     0.00     0.00     0.00
   all   6245   6245    25.75    25.75     1.00     1.00     1.00
Structure 71: https://doi.org/S0108768109026974 i
 order nref-all nref-gt i/s-all i/s-gt    %int-all %int-gt   falloff-fract
     0   7597   5210    13.79    19.63     0.82     0.82     1.00
     1   7907   6958     9.05    10.10     0.18     0.18     0.22
   all  15504  12168    11.37    14.18     1.00     1.00     1.00
Structure 72: https://doi.org/S0108768108016029 i
 order nref-all nref-gt i/s-all i/s-gt    %int-all %int-gt   falloff-fract
     0    318    311    33.42    34.16     0.94     0.94     1.00
     1    512    429     9.89    11.60     0.06     0.06     0.07
   all    830    740    18.90    21.08     1.00     1.00     1.00
Structure 73: https://doi.org/S2052520614007707 o
 order nref-all nref-gt i/s-all i/s-gt    %int-all %int-gt   falloff-fract
     0   2184   2077    23.90    25.08     0.86     0.87     1.00
     1   4258   3540     9.87    11.66     0.13     0.13     0.15
     2   3398   1297     2.16     4.16     0.01     0.00     0.01
   all   9840   6914    10.32    14.29     1.00     1.00     1.00
Structure 74: https://doi.org/S2052520615011713 i
 order nref-all nref-gt i/s-all i/s-gt    %int-all %int-gt   falloff-fract
     0    388    366    81.74    86.59     0.98     0.99     1.00
     1    770    187     1.56     3.78     0.02     0.01     0.02
   all   1158    553    28.43    58.59     1.00     1.00     1.00
Structure 75: https://doi.org/S2052520619014938 o
 order nref-all nref-gt i/s-all i/s-gt    %int-all %int-gt   falloff-fract
     0  10310   8592    19.98    23.78     0.35     0.35     1.00
     1  20321  16589    14.25    17.23     0.25     0.25     0.71
     2  20178  16012    11.79    14.60     0.16     0.16     0.45
     3  20213  16071    12.41    15.35     0.18     0.18     0.53
     4  10016   7751     9.99    12.62     0.06     0.06     0.17
   all  81038  65015    13.38    16.43     1.00     1.00     1.00
Structure 76: https://doi.org/S2052520614011676 (a) i
 order nref-all nref-gt i/s-all i/s-gt    %int-all %int-gt   falloff-fract
```

0	1172	1159	56.50	57.12	0.98	0.98	1.00
1	2213	1845	4.97	5.74	0.02	0.02	0.02
all	3385	3004	22.81	25.56	1.00	1.00	1.00

Structure 77: https://doi.org/S2052520614011676 (b) i

order	nref-all	nref-gt	i/s-all	i/s-gt	%int-all	%int-gt	falloff-fract
0	754	754	56.92	56.92	0.98	0.98	1.00
1	2821	2072	4.15	5.24	0.02	0.02	0.02
all	3575	2826	15.28	19.03	1.00	1.00	1.00

Structure 78: https://doi.org/S0108768106001510 i

order	nref-all	nref-gt	i/s-all	i/s-gt	%int-all	%int-gt	falloff-fract
0	1999	1930	20.14	20.83	0.97	0.97	1.00
1	5448	4471	9.37	11.19	0.03	0.03	0.03
all	7447	6401	12.26	14.10	1.00	1.00	1.00

Structure 79: https://doi.org/S2052520619002105 i

order	nref-all	nref-gt	i/s-all	i/s-gt	%int-all	%int-gt	falloff-fract
0	897	892	40.27	40.49	0.99	0.99	1.00
1	1528	1012	4.22	5.78	0.01	0.01	0.01
all	2425	1904	17.55	22.05	1.00	1.00	1.00

Structure 80: https://doi.org/S0108768109026986 i

order	nref-all	nref-gt	i/s-all	i/s-gt	%int-all	%int-gt	falloff-fract
0	1332	1130	20.02	23.41	0.98	0.99	1.00
1	2444	526	2.00	6.84	0.02	0.01	0.02
all	3776	1656	8.36	18.14	1.00	1.00	1.00

Structure 81: https://doi.org/S2052252524008583 mo

order	nref-all	nref-gt	i/s-all	i/s-gt	%int-all	%int-gt	falloff-fract
0	682	679	178.85	179.63	0.83	0.83	1.00
1	1217	1139	39.24	41.84	0.16	0.16	0.19
2	1098	527	5.57	10.55	0.01	0.01	0.01
all	2997	2345	58.67	74.70	1.00	1.00	1.00

Structure 82: Structure 2 of this paper o

order	nref-all	nref-gt	i/s-all	i/s-gt	%int-all	%int-gt	falloff-fract
0	3112	1728	6.34	10.74	0.32	0.33	1.00
1	6145	3524	6.44	10.62	0.38	0.39	1.19
2	6097	3242	5.68	9.95	0.18	0.18	0.56
3	5905	2733	4.37	8.52	0.08	0.08	0.25
4	5418	1968	2.95	6.75	0.04	0.03	0.11
all	26677	13195	5.09	9.46	1.00	1.00	1.00

Structure 83: Structure 1 of this paper o

order	nref-all	nref-gt	i/s-all	i/s-gt	%int-all	%int-gt	falloff-fract
0	2494	2020	15.73	19.16	0.46	0.46	1.00
1	5018	4062	12.69	15.41	0.35	0.35	0.77
2	4986	3798	7.48	9.48	0.14	0.14	0.30
3	4570	2602	3.82	5.94	0.05	0.04	0.10
all	17068	12482	9.24	12.24	1.00	1.00	1.00

Structure 84: https://doi.org/S0108768103027605 o

order	nref-all	nref-gt	i/s-all	i/s-gt	%int-all	%int-gt	falloff-fract
0	3648	2155	7.33	11.90	0.54	0.56	1.00
1	7493	4507	6.58	10.42	0.33	0.33	0.61
2	7532	3337	3.66	7.37	0.09	0.08	0.17
3	7567	2118	1.86	5.01	0.04	0.03	0.07
all	26240	12117	4.48	8.90	1.00	1.00	1.00

Structure 85: https://doi.org/10.1039-d3ce00119a mo

order	nref-all	nref-gt	i/s-all	i/s-gt	%int-all	%int-gt	falloff-fract
0	1410	830	9.08	14.85	0.87	0.89	1.00

```
     1   2175    763     2.66     6.04     0.13     0.11     0.15
   all   3585   1593     5.18    10.63     1.00     1.00     1.00
Structure 86: https://doi.org/10.1021-acs.jpclett.3c00215 mo
 order nref-all nref-gt i/s-all i/s-gt    %int-all %int-gt   falloff-fract
     0    942    837    38.38    43.05     0.96     0.97     1.00
     1   3465   2068     3.79     5.66     0.04     0.03     0.04
   all   4407   2905    11.18    16.43     1.00     1.00     1.00
Structure 87: https://doi.org/10.1002-chem.202104151 mo
 order nref-all nref-gt i/s-all i/s-gt    %int-all %int-gt   falloff-fract
     0  29514  27732    14.10    14.93     0.92     0.93     1.00
     1  47238  31040     7.04    10.24     0.07     0.07     0.08
     2  47625   8454     1.24     4.15     0.01     0.00     0.01
   all 124377  67226     6.49    11.41     1.00     1.00     1.00
Structure 88: https://doi.org/10.1021-ic701962b i
 order nref-all nref-gt i/s-all i/s-gt    %int-all %int-gt   falloff-fract
     0   5008   4929    24.42    24.79     0.89     0.89     1.00
     1   5485   5327    12.08    12.40     0.11     0.11     0.12
   all  10493  10256    17.97    18.36     1.00     1.00     1.00
Structure 89: https://doi.org/10.1021-jacs.9b02599 i
 order nref-all nref-gt i/s-all i/s-gt    %int-all %int-gt   falloff-fract
     0    790    715    80.95    89.34     0.96     0.97     1.00
     1   1413    430     3.70    10.23     0.04     0.03     0.04
   all   2203   1145    31.40    59.63     1.00     1.00     1.00
Structure 90: https://doi.org/10.1021-ic402930d i
 order nref-all nref-gt i/s-all i/s-gt    %int-all %int-gt   falloff-fract
     0    648    550    24.16    28.28     0.96     0.96     1.00
     1    963    479     4.78     8.76     0.04     0.04     0.04
     2    703     29     0.68     5.40     0.00     0.00     0.00
   all   2314   1058     8.96    18.82     1.00     1.00     1.00
Structure 91: https://doi.org/10.1021-acs.inorgchem.6b01233 i
 order nref-all nref-gt i/s-all i/s-gt    %int-all %int-gt   falloff-fract
     0   3768   2690    17.95    24.81     0.95     0.95     1.00
     1   5958   2721     3.61     7.18     0.05     0.05     0.06
   all   9726   5411     9.17    15.94     1.00     1.00     1.00
Structure 92: https://doi.org/10.1021-ic1008895a i
 order nref-all nref-gt i/s-all i/s-gt    %int-all %int-gt   falloff-fract
     0    989    733    34.57    46.38     0.97     0.98     1.00
     1   1410    398     1.73     4.55     0.02     0.02     0.02
     2    961     32     0.56     4.05     0.00     0.00     0.00
   all   3360   1163    11.06    30.90     1.00     1.00     1.00
Structure 93: https://doi.org/10.1021-ic1008895b i
 order nref-all nref-gt i/s-all i/s-gt    %int-all %int-gt   falloff-fract
     0   3436   3018     6.47     7.23     0.95     0.96     1.00
     1   4678   1446     1.75     3.98     0.05     0.04     0.05
   all   8114   4464     3.75     6.18     1.00     1.00     1.00
Structure 94: https://doi.org/10.1021-jacs.0c08794 mo
 order nref-all nref-gt i/s-all i/s-gt    %int-all %int-gt   falloff-fract
     0    322    189     5.00     7.77     0.66     0.69     1.00
     1    946    517     4.31     7.09     0.22     0.22     0.34
     2   1970    439     1.66     4.56     0.11     0.08     0.17
   all   3238   1145     2.76     6.23     1.00     1.00     1.00
Structure 95: https://doi.org/10.1021-cm402729r i
 order nref-all nref-gt i/s-all i/s-gt    %int-all %int-gt   falloff-fract
     0    406    406   152.77   152.77     0.99     0.99     1.00
```

1	51	51	72.20	72.20	0.01	0.01	0.01
all	457	457	143.78	143.78	1.00	1.00	1.00

Structure 96: https://doi.org/10.1021-ic4013608 i

order	nref-all	nref-gt	i/s-all	i/s-gt	%int-all	%int-gt	falloff-fract
0	1727	1311	9.84	12.62	0.94	0.95	1.00
1	2985	1140	2.23	4.27	0.06	0.05	0.06
all	4712	2451	5.02	8.74	1.00	1.00	1.00

Structure 97: https://doi.org/10.1021-ja206441x i

order	nref-all	nref-gt	i/s-all	i/s-gt	%int-all	%int-gt	falloff-fract
0	637	637	35.08	35.08	0.92	0.92	1.00
1	1531	1531	13.63	13.63	0.07	0.07	0.08
2	51	51	4.89	4.89	0.00	0.00	0.00
all	2219	2219	19.59	19.59	1.00	1.00	1.00

Structure 98: https://doi.org/10.1021-acs.inorgchem.6b02241 i

order	nref-all	nref-gt	i/s-all	i/s-gt	%int-all	%int-gt	falloff-fract
0	3420	3165	20.65	22.22	0.81	0.82	1.00
1	6630	5191	8.04	9.99	0.18	0.18	0.22
2	4819	721	1.15	3.35	0.01	0.00	0.01
all	14869	9077	8.71	13.73	1.00	1.00	1.00

Structure 99: https://doi.org/10.1021-acs.inorgchem.9b00408 i

order	nref-all	nref-gt	i/s-all	i/s-gt	%int-all	%int-gt	falloff-fract
0	2445	2202	28.22	31.22	0.88	0.88	1.00
1	1298	955	18.31	24.64	0.09	0.08	0.10
2	1320	808	8.67	13.74	0.03	0.03	0.04
3	1265	482	3.00	6.91	0.01	0.01	0.01
all	6328	4447	17.07	24.00	1.00	1.00	1.00

Structure 100: https://doi.org/10.1021-ic5015412 i

order	nref-all	nref-gt	i/s-all	i/s-gt	%int-all	%int-gt	falloff-fract
0	411	325	36.67	46.11	0.92	0.92	1.00
1	1641	1326	7.62	9.19	0.08	0.08	0.09
all	2052	1651	13.44	16.46	1.00	1.00	1.00

Structure 101: https://doi.org/10.1021-acs.cgd.9b01348 mo

order	nref-all	nref-gt	i/s-all	i/s-gt	%int-all	%int-gt	falloff-fract
0	3476	1817	11.28	20.83	0.75	0.78	1.00
1	6768	3068	5.06	10.15	0.22	0.21	0.30
2	2831	575	1.77	5.84	0.03	0.02	0.03
all	13075	5460	6.00	13.25	1.00	1.00	1.00

Structure 102: https://doi.org/10.1021-ja993532s i

order	nref-all	nref-gt	i/s-all	i/s-gt	%int-all	%int-gt	falloff-fract
0	201	201	33.79	33.79	0.40	0.40	1.00
1	156	156	25.90	25.90	0.16	0.16	0.41
2	245	245	27.14	27.14	0.24	0.24	0.59
3	111	111	23.35	23.35	0.09	0.09	0.22
4	96	96	21.36	21.36	0.07	0.07	0.19
5	38	38	20.68	20.68	0.03	0.03	0.06
6	15	15	28.88	28.88	0.01	0.01	0.03
7	2	2	25.25	25.25	0.00	0.00	0.00
all	864	864	27.07	27.07	1.00	1.00	1.00

Structure 103: https://doi.org/10.1021-acs.inorgchem.0c01722 i

order	nref-all	nref-gt	i/s-all	i/s-gt	%int-all	%int-gt	falloff-fract
0	690	671	72.03	74.04	0.79	0.79	1.00
1	1213	1074	24.33	27.34	0.20	0.20	0.25
2	1029	443	4.86	9.99	0.02	0.02	0.02
all	2932	2188	28.73	38.14	1.00	1.00	1.00

Structure 104: https://doi.org/10.2138-am-2017-5807 i

order	nref-all	nref-gt	i/s-all	i/s-gt	%int-all	%int-gt	falloff-fract
0	2025	1883	68.23	73.31	0.89	0.89	1.00
1	3912	2882	13.32	17.79	0.11	0.10	0.12
2	3522	1032	1.94	4.92	0.01	0.00	0.01
all	9459	5797	20.84	33.53	1.00	1.00	1.00

Structure 105: https://doi.org/S0108768109029565 i

order	nref-all	nref-gt	i/s-all	i/s-gt	%int-all	%int-gt	falloff-fract
0	693	659	34.04	35.75	0.98	0.98	1.00
1	1324	577	3.70	7.41	0.02	0.02	0.02
all	2017	1236	14.12	22.52	1.00	1.00	1.00

Structure 106: https://doi.org/S0108768107021647 i

order	nref-all	nref-gt	i/s-all	i/s-gt	%int-all	%int-gt	falloff-fract
0	1118	939	30.72	36.44	0.87	0.88	1.00
1	4419	2193	3.86	7.10	0.13	0.12	0.15
all	5537	3132	9.28	15.90	1.00	1.00	1.00

Structure 107: https://doi.org/S2052520623005292 i

order	nref-all	nref-gt	i/s-all	i/s-gt	%int-all	%int-gt	falloff-fract
0	974	934	46.26	48.18	0.99	0.99	1.00
1	963	366	2.74	6.32	0.01	0.01	0.01
all	1937	1300	24.62	36.40	1.00	1.00	1.00

Structure 108: https://doi.org/10.1021-acs.cgd.4c01457 mo

order	nref-all	nref-gt	i/s-all	i/s-gt	%int-all	%int-gt	falloff-fract
0	4240	3214	18.85	24.57	0.60	0.61	1.00
1	8416	6266	16.91	22.41	0.31	0.31	0.52
2	8138	5103	9.05	13.89	0.07	0.07	0.12
3	7065	2242	2.35	5.70	0.01	0.01	0.02
all	27859	16825	11.22	18.01	1.00	1.00	1.00

Structure 109: https://doi.org/10.1021-acs.cgd.1c01429 o

order	nref-all	nref-gt	i/s-all	i/s-gt	%int-all	%int-gt	falloff-fract
0	1833	1183	18.01	27.38	0.62	0.63	1.00
1	3757	2544	19.51	28.35	0.32	0.32	0.51
2	3367	1483	4.22	8.40	0.06	0.05	0.09
all	8957	5210	13.46	22.45	1.00	1.00	1.00

Structure 110: https://doi.org/10.1021-jacs.5b08230 i

order	nref-all	nref-gt	i/s-all	i/s-gt	%int-all	%int-gt	falloff-fract
0	2834	2750	24.31	25.02	0.96	0.96	1.00
1	4741	4429	18.53	19.74	0.04	0.04	0.05
all	7575	7179	20.69	21.76	1.00	1.00	1.00

Structure 111: https://doi.org/10.1515-zkri-2016-2013 mo

order	nref-all	nref-gt	i/s-all	i/s-gt	%int-all	%int-gt	falloff-fract
0	2992	2955	68.06	68.90	0.73	0.73	1.00
1	5933	5690	36.51	38.02	0.22	0.22	0.30
2	5688	5018	15.70	17.66	0.05	0.05	0.07
all	14613	13663	34.87	37.22	1.00	1.00	1.00

Structure 112: https://doi.org/10.1080-08957959.2024.2444685 i

order	nref-all	nref-gt	i/s-all	i/s-gt	%int-all	%int-gt	falloff-fract
0	953	950	30.77	30.87	0.98	0.98	1.00
1	739	372	4.69	8.71	0.02	0.02	0.02
2	919	246	2.05	6.21	0.00	0.00	0.00
all	2611	1568	13.28	21.74	1.00	1.00	1.00

Structure 113: https://doi.org/10.1103-PhysRevB.108.134104 mo

order	nref-all	nref-gt	i/s-all	i/s-gt	%int-all	%int-gt	falloff-fract
0	4184	3784	17.28	18.98	0.94	0.94	1.00

```
     1   7581   4450     3.19     4.66     0.06     0.06     0.07
   all  11765   8234     8.20    11.24     1.00     1.00     1.00
Structure 114: https://doi.org/10.1021-acs.chemmater.4c00925 i
 order nref-all nref-gt i/s-all i/s-gt    %int-all %int-gt   falloff-fract
     0   1924   1742    31.61    34.83     1.00     1.00     1.00
     1   7292    364     0.52     3.10     0.00     0.00     0.00
   all   9216   2106     7.01    29.35     1.00     1.00     1.00
Structure 115: https://doi.org/10.1021-acs.cgd.9b00518 o
 order nref-all nref-gt i/s-all i/s-gt    %int-all %int-gt   falloff-fract
     0   5126   5068    12.70    12.82     0.95     0.95     1.00
     1   2615   2496     4.96     5.13     0.04     0.04     0.05
     2    737    651     4.48     4.89     0.01     0.01     0.01
   all   8478   8215     9.60     9.86     1.00     1.00     1.00
Structure 116: https://doi.org/10.1088-2515-7639-ab423b mo
 order nref-all nref-gt i/s-all i/s-gt    %int-all %int-gt   falloff-fract
     0   2159   2012    11.83    12.60     0.78     0.78     1.00
     1   4231   3558     6.15     7.09     0.22     0.22     0.28
   all   6390   5570     8.07     9.08     1.00     1.00     1.00
Structure 117: https://doi.org/S1600576723002261 i
 order nref-all nref-gt i/s-all i/s-gt    %int-all %int-gt   falloff-fract
     0    446    446    38.82    38.82     0.94     0.94     1.00
     1    377    317    22.92    27.14     0.05     0.05     0.06
     2    383    170     3.18     6.15     0.00     0.00     0.00
     3    348    157     2.76     5.35     0.00     0.00     0.00
   all   1554   1090    18.10    25.51     1.00     1.00     1.00
-----------------------------------------------
50% percentile:
11.362330875329462
90% percentile:
48.96277656887369
Counts of each type:
{'h': 2, 'i': 85, 'mo': 14, 'o': 16}
```
